# Divergent Metabolic Effects of Metformin Merge to Enhance Eicosapentaenoic Acid Metabolism and Inhibit Ovarian Cancer In Vivo

**DOI:** 10.3390/cancers14061504

**Published:** 2022-03-15

**Authors:** Mary P. Udumula, Laila M. Poisson, Indrani Dutta, Nivedita Tiwari, Seongho Kim, Jasdeep Chinna-Shankar, Ghassan Allo, Sharif Sakr, Miriana Hijaz, Adnan R. Munkarah, Shailendra Giri, Ramandeep Rattan

**Affiliations:** 1 Department of Women’s Health Services, Henry Ford Hospital, Henry Ford Cancer Institute, Detroit, MI 48202, USA; mudumul1@hfhs.org (M.P.U.); ntiwari3@hfhs.org (N.T.); chhinas@frontierscience.org (J.C.-S.); mhijaz1@hfhs.org (M.H.); amunkar1@hfhs.org (A.R.M.); 2 Center for Bioinformatics, Department of Public Health Services, Henry Ford Cancer Institute, Detroit, MI 48202, USA; lpoisso1@hfhs.org (L.M.P.); idatta1@hfhs.org (I.D.); 3 Biostatistics and Bioinformatics Core, Department of Oncology, Karmanos Cancer Institute, Wayne State University, Detroit, MI 48201, USA; kimse@karmanos.org; 4 Department of Pathology, Henry Ford Hospital, Henry Ford Cancer Institute, Detroit, MI 48202, USA; gallo1@hfhs.org; 5 Department of Gynecology Oncology, Barbara Ann Karmanos Cancer Institute, Wayne State University, Detroit, MI 48201, USA; sharif.sakr@ascension.org; 6 Department of Neurology, Henry Ford Hospital, Detroit, MI 48202, USA; sgiri1@hfhs.org; 7 Department of Oncology, Wayne State School of Medicine, Detroit, MI 48201, USA

**Keywords:** ovarian cancer, metabolomics, metformin, omega-3 fatty acids, DHA, EPA

## Abstract

**Simple Summary:**

Although the anticancer effects of metformin are well studied, its effect on energy metabolism of cancer cells remains elusive. Metformin can alter the metabolism of cells either by activating AMPK or inhibiting the mitochondrial respiratory chain complex. The aim of the study was to explore the distinct metabolic profiles of ovarian cancer cell lines post-metformin treatment and to understand metformin’s metabolism-based anti-growth effect on ovarian cancer cell lines. In this study, using the untargeted metabolomics approach, we found that metformin treatment promoted omega-3 fatty acid metabolism in three different ovarian cancer cell lines, and treating ovarian xenografts with specific omega-3 fatty acids, especially EPA, reduced ovarian tumor growth. Thus, our study adds an additional potential therapeutic mechanism to the multi-potent anticancer effects of metformin.

**Abstract:**

Metformin is being actively repurposed for the treatment of gynecologic malignancies including ovarian cancer. We investigated if metformin induces analogous metabolic changes across ovarian cancer cells. Functional metabolic analysis showed metformin caused an immediate and sustained decrease in oxygen consumption while increasing glycolysis across A2780, C200, and SKOV3ip cell lines. Untargeted metabolomics showed metformin to have differential effects on glycolysis and TCA cycle metabolites, while consistent increased fatty acid oxidation intermediates were observed across the three cell lines. Metabolite set enrichment analysis showed alpha-linolenic/linoleic acid metabolism as being most upregulated. Downstream mediators of the alpha-linolenic/linoleic acid metabolism, eicosapentaenoic acid (EPA) and docosahexaenoic acid (DHA), were abundant in all three cell lines. EPA was more effective in inhibiting SKOV3 and CaOV3 xenografts, which correlated with inhibition of inflammatory markers and indicated a role for EPA-derived specialized pro-resolving mediators such as Resolvin E1. Thus, modulation of the metabolism of omega-3 fatty acids and their anti-inflammatory signaling molecules appears to be one of the common mechanisms of metformin’s antitumor activity. The distinct metabolic signature of the tumors may indicate metformin response and aid the preclinical and clinical interpretation of metformin therapy in ovarian and other cancers.

## 1. Introduction

Ovarian cancer is a challenge, being the gynecologic cancer with the highest mortality, albeit a low incidence rate [1]. Thus, finding new or repurposed therapeutics and approaches for ovarian cancer is urgently required. Metformin, the most prescribed antidiabetic drug for type 2 diabetes has been and is being actively pursued as a repurposed drug in various cancers, including ovarian cancer. Metformin has inhibitory effects on almost every type of cancer, including ovarian cancer [2,3,4,5,6]. Retrospective and epidemiological studies have well documented the benefits of metformin intake in reducing cancer incidence and improving outcomes in ovarian cancer [7,8,9,10]. Many of the past and current clinical trials support the potential of metformin in combination with standard chemotherapy [11,12]. Currently, 20 trials are ongoing and recruiting patients for various cancers, such as breast, bladder, endometrial and ovarian cancer (CliniclTrials.gov; accessed on 15 December 2021).

Although the anticancer effects of metformin are well documented, the exact underlying mechanisms are still unclear. The most established is the activation of the master metabolism regulator 5′ AMP-activated protein kinase (AMPK) due to the energy imbalance created by inhibition of the complex 1 activity in the mitochondria [13,14,15]. AMPK activation leads to downregulation of multiple oncogenic pathways controlling cell proliferation, such as the Alpha serine/threonine protein kinase (Akt), mammalian target of rapamycin (mTOR), Hypoxia-inducible factor 1-alpha (HIF-1), and Nuclear factor kappa light chain enhancer of activated B cells (NFkB), and tyrosine kinase pathways, such as epidermal growth factor, and ErbB2 and WNT/β-catenin signaling [16,17]. Through activation of AMPK, metformin also regulates insulin signaling, whole body energy balance, and the metabolism of glucose and lipids [18]. AMPK-independent anti-tumor effects of metformin have been reported, which include inhibition of mTOR by regulating Rag GTPases independent of AMPK [19], modulation of miRNA [20], and inhibition of the UPR and Wnt/ β-Catenin pathway in endometrial cancer [21]. Overall, metformin has significant potential as an anticancer drug that functions both in an AMPK-dependent and -independent manner.

Although the mechanism of action of metformin is debatable, one of the certain downstream effects of metformin includes the change in energy metabolism of cells, either as a consequence of its glucose-lowering effect or by activation of AMPK and other AMP-sensitive cellular components [22,23,24], or by its inhibitor effects on the mitochondrial respiratory chain complex 1 [25], which affects the metabolic profile of cells [18]. The metabolomic profile of metformin action in type 2 diabetic patients indicated altered glucose, ketone body, and fatty acid metabolism [26]. Metformin treatment induced lipid and glycogen metabolism in serum and tumors of endometrial cancer patients [27] and induced ketone body, β-hydroxy butyrate, and TCA cycle intermediates in breast cancer patients [28]. Metabolomic studies in metformin-treated ovarian cancer patients also showed an extensive remodeling of metabolism by inhibiting the electron transport chain, a decrease in short-chain acyl carnitines, and an increase in NADH, but all these changes were seen in the presence of a glucose-rich medium. In nutrient-limited conditions, adaptive pathways such as fatty acid oxidation were increased [29,30,31].

The objective of our study was to identify the common global metabolic profiles of three ovarian cancer cell lines (A2780, A2780 cisplatin-resistant derivative C200, and the highly aggressive cell line SKOV3ip) following treatment with metformin to understand metformin’s metabolism-based anti-growth effect on ovarian cancer cell lines. Our study demonstrates that metformin commonly alters the essential fatty acid metabolism by promoting the metabolism of omega-3 fatty acids, specifically eicosapentaenoic acid (EPA). Thus, our study adds an additional potential therapeutic mechanism to the multi-potent anticancer effects of metformin that can be exploited to design new combination approaches to provide metformin’s maximum benefit in various cancers.

## 2. Materials and Methods

### 2.1. Cell Lines and Media

Human ovarian cancer cells A2780 and C200 cell lines were a kind gift from Dr. Thomas Hamilton, Fox Chase Cancer Center, PA, USA [32]. The cell lines were maintained in Roswell Park Memorial Institute media (RPMI 1640; HyClone-Thermo Scientific, Waltham, MA, USA) supplemented with 10% fetal bovine serum (FBS; BioAbChem, Ladson, SC, USA), insulin (Henry Ford Pharmacy, Detroit, MI, USA), and 100 units/mL penicillin and 100 µg/mL streptomycin at 37 °C under 5% CO_2_. CaOV3 (HTB-75) cells were purchased from ATCC (Manassas, VA, USA) and grown in RPMI 1640 media containing 10% FBS, 100 units/mL penicillin, 100 µg/mL streptomycin, and 1500 mg/L of sodium bicarbonate. SKOV3ip cells were a kind gift from the laboratory of Drs. Mien-Chie Hung and Yu Dihua (MD Anderson Cancer Center, Houston, TX, USA) [33] and grown in McCoy’s 5A media supplemented with 10% FBS, 100 units/mL penicillin, and 100 µg/mL streptomycin. For in vitro purpose, cells were seeded and treated with metformin and fatty acids (FAs) under serum-free conditions. FAs docosahexaenoic acid (DHA) and EPA (Cayman Chemical Company, Ann Arbor, MI, USA) were diluted in ethanol, with final concentration of ethanol (<0.1%) in treated cultures. For animal studies, DHA and EPA were diluted in corn oil.

### 2.2. Seahorse Metabolic Analysis

A2780, C200, and SKOV3ip cells were plated in RPMI 1640 at a density of 6 × 10^5^ cells/well in cell-tak-coated XFe 96-cell plates. Oxidative phosphorylation (OCR) and extracellular acidification (ECAR) rate were measured using an XFe 96 Seahorse Analyzer (Agilent Seahorse XF Analyzers, Santa Clara, CA, USA) as described previously [34,35]. OCR measurements were recorded with port injections of (1) oligomycin (1 µmol), (2) carbonyl cyanide-4 (trifluoromethoxy) phenylhydrazone (FCCP) (0.5 µmol), and a combination of (3) rotenone-antimycin (1 µmol). ECAR measurements were recorded after injecting with (1) glucose (10 mM), followed by (2) oligomycin (2 µM), and (3) 2-Deoxy-D-glucose (2DG) (100 mM). The measurements were normalized with the cell number. Basal OCR was calculated as the average of the first 3 measurements subtracted by the OCR measurements after rotenone and antimycin D injections, and indicates the baseline of respiration of the cell in the presence of glucose. Basal ECAR was calculated as ECAR in the presence of glucose after subtracting the ECAR after 2DG injection, when glucose was given as fuel [34].

### 2.3. Cell Proliferation Assays

The MTT colorimetric assay was used to measure cell growth and viability as described previously [34]. Briefly, A2780, C200, SKOV3ip, and CaOV3 cells (1.2 × 10^4^ cells/well) were seeded in a flat-bottomed 96-well plate and incubated with either solvent control (0.5% ethanol) or various concentrations of DHA or EPA for different periods of time. After incubation, the relative cell viability was determined by the MTT assay and recorded by a microplate reader.

### 2.4. Metabolomics

#### 2.4.1. Metabolite Assessment

Cells were prepared by growing in 100 mM plates (*n* = 10/cell line) using RPMI with 10% FBS for all the cell lines. One set (*n* = 5) was treated with 10 mM of metformin for 24 h, after which 10 × 10^6^ cells were counted, washed with phosphate buffered saline (PBS), and snap frozen per vial for untreated (*n* = 5) and treated (*n* = 5) cells from each cell line. The cells were shipped to Metabolon Inc. (Durham, NC, USA) for untargeted metabolomic profiling, as previously described [19,20,21,22].

#### 2.4.2. Data Analysis

To control for sample concentration, each metabolite intensity value was standardized as a ratio against the Bradford protein measure for that sample. Missing values that indicated a limit of detection by the mass spectrometer were replaced with a small value (the study minimum) for analysis. Each metabolite was scaled to have a median of 1.0 across all samples. Metabolite profiles by line and treatment were visualized by principal component analysis. Metabolite intensities (log2) were compared between lines and treatments using two-sample *t*-tests comparing treated and control samples within a line. False discovery rates were estimated by conversion of the *p*-value to a *q*-value. FDR was estimated separately for each treated-control comparison. Changes determined from the per-metabolite *t*-tests (FDR < 0.05) were classified according to direction of change from control, i.e., up, down, and unchanged. Heatmaps and boxplots were drawn to represent metabolite level changes in specific pathways related to the energetics hypothesis, e.g., glycolysis, TCA cycle, long- and medium-chain fatty acid biosynthesis, and omega-3 and omega-6 fatty acid biosynthesis. 

#### 2.4.3. Pathway Analysis

Metabolite profile changes were also compared across the 3 cell lines. All metabolites with a significant change after treatment, in any line (*t*-test, FDR < 0.05) were drawn in a heatmap, split by broad metabolite class, e.g., carbohydrate, lipid. Intensity was scaled, by per metabolite mean and standard deviation. Next, we explored the set of metabolites that were changed by treatment in each of the three lines. We performed pathway analysis separately on those metabolites consistently upregulated (increased in all three lines) and consistently downregulated (decreased in all three lines) after treatment. Two pathway analysis database tools were applied. First, metabolite set enrichment analysis (MSEA; MetaboAnalyst 5.0; last accessed March 2022) was used to perform over-representation analysis to evaluate whether the set of significantly altered metabolites was represented more than expected by chance with the human SMDB pathways. The MetaboAnalyst ID Conversion tool was used to update the biochemical naming and HMDB ID numbers, and compound names were used to enter the changed metabolite lists for MSEA. Bar graphs indicating the enrichment ratio were exported for those pathways for which *p* ≤ 0.05. Asterisks are added to the bars where FDR < 0.05. Second, network construction was performed using the Ingenuity Pathway Analysis (IPA) core analysis of metabolites (QIAGEN, Redwood City, CA, USA; last accessed March 2022). IPA is based on the Biocyc pathways and the proprietary Ingenuity knowledgebase. The Human Metabolon Database identifiers were used to map the metabolites to the IPA knowledgebase. Interactions from both experimentally validated and high-confidence predictions were used. The top generated pathways for the consistently upregulated and consistently downregulated metabolites were exported. Enrichment of canonical pathways and prediction of upstream regulators were also considered.

### 2.5. Mouse Studies

#### 2.5.1. Ethics Statement

All protocols were approved by the Henry Ford Hospital Institutional Animal Care and Use Committee prior to any experiments. All institutional and national guidelines for the care and use of laboratory animals were followed.

#### 2.5.2. Tumor Inoculation and Treatment

Female nude mice of 6–7 weeks age were obtained from Jackson Laboratory (Bar Harbor, ME, USA) and used according to the approved protocol by the animal care and use committee. Cells were washed and resuspended in PBS at 3 × 10^6^ cells/200 µL. SKOV3ip and CaOV3 cells were injected in the peritoneal cavity of each mouse. One week after inoculation, mice were randomly grouped into control (vehicle corn oil, 100 mL), DHA, or EPA. Mice were dosed orally with EPA and DHA 100 mg/kg body weight in corn oil, for 5 days per week, for a total of 4–5 weeks. Body weight and tumor growth was monitored once a week until the end of the study. Autopsies were performed at week 4 for SKOV3 and week 5 for CaOV3 xenografts.

### 2.6. Total RNA Isolation and Real-Time PCR

Total RNA was extracted from liver and tumor tissues using an RNA assay kit (Qiagen, Valencia, CA, USA) and quantified by Qubit Fluorometer (Invitrogen, Waltham, MA, USA). Reverse transcription was performed using 1 µg of total RNA using a high-capacity cDNA kit in a 20 µL reaction mixture and real-time polymerase chain reactions were performed as previously described [35] in a 25 µL mixture (1 µL complementary DNA, 1 × SYBR green buffer, forward and reverse specified primers). Real-time quantifications were performed using CFX Bio-Rad Laboratories Real-Time Polymerase Chain Reaction Detection system (Hercules, CA, USA). Ribosomal protein L27 was used as a housekeeping gene. All primers were purchased from Integrated DNA Technologies (Coralville, IA, USA) (Table S1).

### 2.7. Western Blot Analysis

After treatment, protein was isolated from tumors of both xenograft models by a protein lysis buffer (50 mM Tris-HCL, Ph 7.5, 250 mM NaCl, 5 mM EDTA, 50 mM NaF, and 0.5% Nonidet P-40, containing a protease inhibitor cocktail (Sigma, St Louis, MO, USA). Protein was quantified using a bicinchoninic acid assay kit (Thermo Fisher) and equal protein was loaded and separated by 12% sodium dodecyl-sulfate (SDS) polyacrylamide gel electrophoresis, transferred onto a polyvinylidene difluoride membrane, and blocked with 5% skim milk. Target proteins such as phospho-AMPK (pAMPK), phospho-acetyl Co A carboxylase alpha (pACC), total-AMPK (t-AMPK), total ACC (t-ACC), cyclin D1, p21, cleaved caspase-3, cleaved PARP, Bcl-xL, and peroxisome proliferator receptor activator (PPARγ, lipoxygenases (5-LOX and 15-LOX) were immunodetected as previously described [35]. Dilutions for the antibodies are stated in Table S2.

### 2.8. Enzyme-Linked Immunosorbent Assay

Protein levels of interleukin (IL)-6, IL-1β, Monocyte Chemoattractant Protein (MCP)-1, and Tumor Necrosis Factor (TNF)-α (Biolegend enzyme-linked immunosorbent assay kits, San Diego, CA, USA), and Resolvin E1 (RVE1) and Resolvin D1 (RVD1) (MyBiosource, San Diego, CA, USA), were measured in plasma. Enzyme-linked immunosorbent assays (ELISA) were performed according to manufacturer’s instructions. Exact kits are enumerated in Table S3.

### 2.9. Cell Cycle Analysis

The cell cycle profiles of A2780, C200, SKOV3ip, and CaOV3 cells were analyzed as described previously [4] by staining the DNA content in the cells. A2780, C200, SKOV3ip, and CaOV3 (1.5 × 10^5^ cells/dish) were seeded and treated the next day with solvent control (0.5% ethanol), DHA and EPA for 48 h. For analysis, the cells were permeabilized with 70% ethanol at 4 °C for 30 min. Subsequently, the cells were treated with propidium iodide and analyzed by flow cytometry (BD BioSciences, San Jose, CA, USA) for cell cycle distribution using the ModFit LT V3.0 software (Verity Software House, Topsham, ME, USA).

### 2.10. Hoechst Staining

A2780, C200, SKOV3ip, and CaOV3 cells were cultured on cover slips and treated with DHA and EPA for 48 h. After, cells were washed and incubated in Hoechst buffer (Hank’s balanced salt solution, 10% fetal calf serum, 20 mM HEPES (4-(2-hydroxyethyl)-1-piperazineethanesulfonic acid) and 1% D-glucose) and stained with 5 µm of Hoechst dye (Thermo Fischer Scientific) for 30 min at 37 °C [36]. The percentage fragmentation index was measured by counting the number of nuclear fragments to the total number of cells in that micrograph.

### 2.11. Immunohistochemistry

Tumors excised from mice were fixed using 10% formalin for 48 h, paraffin embedded, and processed for immunohistochemistry as described previously [5,35]. Immunohistochemistry for p-AMPK (1:100; Cell Signaling Technologies, Denver, MA, USA), Ki67 (1:200; Abcam, Cambridge, MA, USA) and cleaved caspase 3 (1:100; Cell Signaling Technologies) was performed using Dako Autostainer Link 48 (Carpinteria, CA, USA). The slides were examined in a blinded manner by a pathologist under a light microscope and representative images were taken from a minimum of 3 slides from each group. IHC antibodies are listed in Table S4.

### 2.12. TUNEL Assay

Tumor tissue sections were subjected to TUNEL (terminal deoxynucleotidal transferase-mediated biotin-deoxyuridine trisphosphate nick-end labeling) staining (Click-iT TUNEL Colorimetric, Thermo Fischer Scientific) according to the manufacturer’s instructions. Total TUNEL positive cells in the tumor specimens were counted under a light microscope. Five random images were taken under high power magnification. The ratio of total TUNEL positive cells to total tumor cells indicates the apoptosis index [35].

### 2.13. Statistical Methods

Data were plotted and statistically analyzed using two-tailed or unpaired t-tests (GraphPad Prism Software, La Jolla, CA, USA). Survival outcomes were graphically summarized by Kaplan–Meier curves and compared between groups using log-rank tests. Each in vitro experiment was performed a minimum of 3 times with 3 technical replicates.

## 3. Results

### 3.1. Metformin Induces Sustained Effect on Cellular Bioenergetics

**Figure 1:**
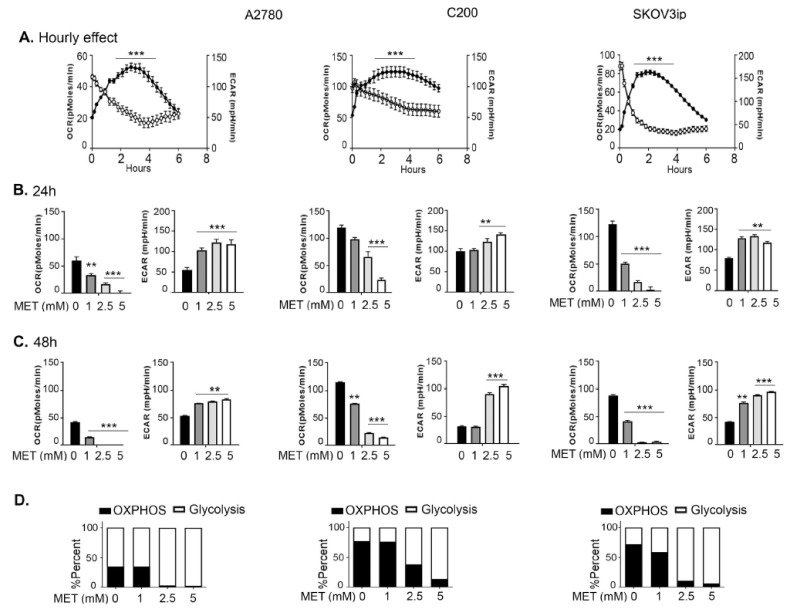
Metformin induces sustained effects on cellular bioenergetics. Cell lines were subjected to increasing metformin (MET) concentrations for increasing time periods and cellular bioenergetics were measured. Basal OCR (oxygen consumption rate) and ECAR (extracellular acidification rate) changes in A2780, C200, and SKOV3ip cells (**A**) after immediate 5 mM metformin exposure, open circles represent OCR and closed circles represent ECAR; (**B**) after 24 h of indicated metformin exposure; (**C**) after 48 h of indicated metformin exposure. (**D**) Bioenergetic profile of the cell lines representing the percent ratio of OCR and ECAR being conducted by each cell line at 24 h of indicated metformin exposure. For all graphs, ** *p* ≤ 0.01, and *** *p* ≤ 0.001, metformin treated compared to untreated.

To test the metabolic effects of metformin, we selected the chemosensitive A2780, its chemoresistant isogeneic C200 derivative, and an aggressive SKOV3 derivative, SKOV3ip human ovarian cancer cell lines [37,38]. Although previously we [4,5] and others [3,6] showed metformin to inhibit the growth of ovarian cancer cell lines, here, we first confirmed the growth inhibitory effect of metformin on A2780, C200, and SKOV3ip cells. Metformin inhibited the growth of all three cell lines in a dose- and time-dependent manner, although A2780 was most sensitive to metformin (Figure S1). For functional metabolomics, we profiled the bioenergetic changes by metformin over time. Metformin induced glycolysis (measured as ECAR) and inhibited oxidative phosphorylation (measured as OCR) in all three cell lines immediately when injected through the injection port of the Seahorse Metabolic Analyzer (Figure 1A). Time course treatments showed that the bioenergetic changes were maintained, as evident from increased ECAR and decreased OCR measurements at 24 and 48 h after metformin treatment (Figure 1B,C). Bioenergetic organization revealed that metformin positioned the cells towards a glycolytic phenotype (Figure 1D). Thus, metformin inhibits ovarian cancer cell line proliferation, which is associated with increased glycolysis and decreased oxidative phosphorylation.

### 3.2. Metformin Induces Differential Cell Line Specific Metabolomic Changes

To test if the metformin-induced bioenergetic changes are reflected in the metabolite profiles, we performed untargeted metabolic profiles of the three cell lines (*n* = 5) after 48 h of metformin treatment (Figure S2A). Principal component analysis (PCA) analysis revealed little intra-sample variability within each cell line with or without metformin treatment (Figure S2B). Interestingly, all three cell lines fell into distinct clusters pre- and post-metformin treatment. Of the 295 metabolites identified by global metabolomics, 233 were altered in at least one cell line (Figure S2D). The heatmap representation of these also reflected the differences in biochemical profiles, where each cell line was drastically different (greater that 70% of the biochemicals) prior to and post-treatment with metformin (Figure S2C). Heat map presentation showed variability across the cell line changes in metabolites, with lipids and amino acids being the largest group of super-metabolic pathways altered by metformin (Figure S2C). A2780 cells showed significant (FDR ≤ 0.05) alterations in 156 metabolites, with 86 increased and 70 decreased after metformin treatment. C200 cells showed significant (FDR ≤ 0.05) alterations in 138 metabolites, with 84 increased and 54 decreased after metformin treatment. SKOV3ip cells showed significant (FDR ≤ 0.05) alterations in 145 metabolites, with 91 increased and 54 decreased after metformin treatment (Figure S2D). Thus, the three ovarian cancer cell lines appear to be metabolically distinct and undergo discrete metabolic alterations in response to metformin.

### 3.3. Changes in Glycolysis Metabolites following Metformin Treatment Were Cell Line Specific

**Figure 2:**
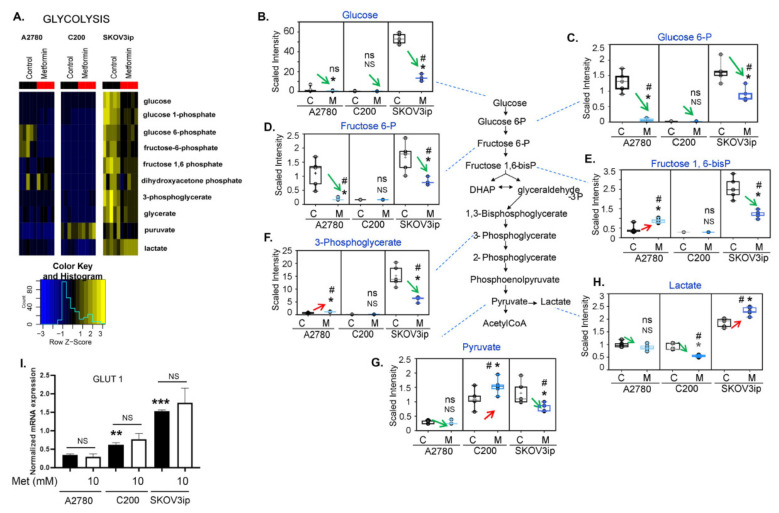
Effect of metformin on glycolysis cycle intermediates is cell-line specific. Selective analysis of glycolysis metabolites from the untargeted metabolomics was performed in untreated control (C) and 10 mM metformin (M)-treated cells. (**A**) Heatmap showing alterations in glycolysis cycle metabolites in A2780, C200, and SKOV3ip cells. Scaled metabolite intensity graphs showing levels of (**B**) glucose, (**C**) glucose-6-phosphate (glucose 6-P), (**D**) fructose 6-phosphate (fructose 6-P), (**E**) fructose 1,6-bisphosphate (fructose 1,6-biP), (**F**) 3-phosphoglycerate, (**G**) pyruvate, and (**H**) lactate in A2780, C200, and SKOV3ip cells in response to metformin (10 mM). For all box-plot graphs, * *p* ≤ 0.05, NS = *p* non-significant, # *q* ≤ 0.05, ns = *q* non-significant when metformin treated compared to control. (**I**) mRNA expression of glucose transporter (GLUT-1) in metformin (10 mM) ** *p* ≤ 0.01 and *** *p* ≤ 0.001, metformin treated compared to untreated.

Glucose on uptake is rapidly converted to glucose-6-phosphate (G6P), which can continue through glycolysis to pyruvate and subsequently enter the TCA cycle as acetyl-coenzyme A (CoA) for energy production or can be shunted into the pentose phosphate pathway to contribute to nucleotide biosynthesis. Metformin treatment significantly altered the glycolytic metabolites in all three cell lines, but the effects were cell-line specific (Figure 2A). SKOV3ip cells had significantly higher glucose levels regardless of treatment (Figure 2B), which may be a result of increased glucose uptake by these cells. Regardless of the different starting levels of glucose, metformin caused a significant decrease in glucose, and the glycolytic intermediates G6P and fructose 6-phosphate (F6P), in both A2780 and SKOV3ip cells (Figure 2C,D). In contrast, although the glycolytic intermediates fructose-1,6-diphosphate (F-1,6-diP) and 3-phosphoglycerate (3PG) were significantly decreased in SKOV3ip, the levels of these two intermediates were significantly increased in A2780 following metformin treatment (Figure 2E,F). It should be noted that, except for 3PG (which was statistically non-significantly elevated in C200 following metformin), G6P, F6P, and F-1,6-diP were not detected in C200 cells (Figure 2C–F). Individual plots for glucose, G6P, and 3PG are included in Supplemental Figure S3 for clarity. Pyruvate was significantly decreased in SKOV3ip and statistically non-significantly decreased in A2780, but was significantly increased in C200 (Figure 2G). In addition to being converted to acetyl CoA, pyruvate can be metabolized to lactate for gluconeogenesis or excretion, and increased conversion of pyruvate to lactate is a typical biochemical pathway that cancer cells utilize for energy production (Warburg metabolism). Although lactate levels were significantly decreased in A2780 and C200 cells following metformin treatment, lactate levels were significantly increased in SKOV3ip (Figure 2H). Thus, all three cell lines appear to initially enhance glycolysis following metformin treatment, but a potential block in the late glycolytic intermediates possibly occurred in A2780 and C200 cells. In contrast, metformin treatment resulted in increased metabolism of glucose and a subsequent increase in pyruvate to lactate in SKOV3ip cells. One possible explanation for the higher glycolytic activity in SKOV3ip may be the higher endogenous levels of glucose due to increased glucose uptake. This is supported by the increased expression of GLUT1 in SKOV3ip cells (Figure 2I). Interestingly, metformin did not alter the expression of GLUT1 in any of the cell lines. Thus, metformin induced an increase in glycolysis activity and the glycolysis metabolite changes do not align clearly. This may suggest that metabolic pathway alterations and metabolic activity are not reflective of each other, and the intricate metabolic crosstalk in conjunction with cell identity may be involved in mediating metformin’s effects.

### 3.4. Changes in TCA Metabolites following Metformin Treatment Were Cell-Line Specific

**Figure 3:**
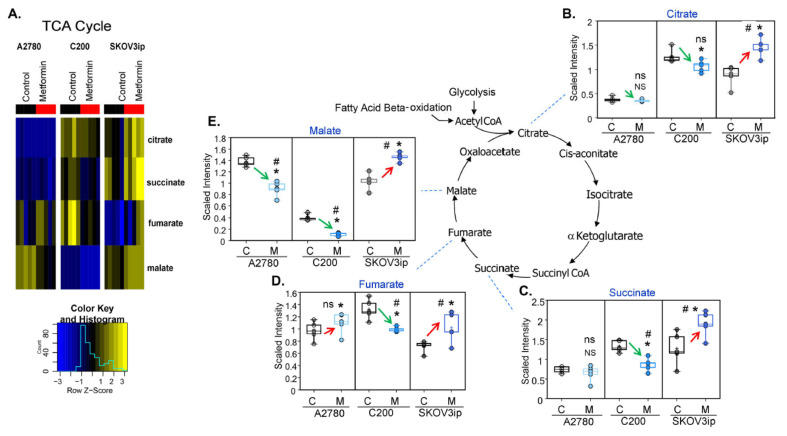
Effect of metformin on TCA cycle intermediates is cell-line specific. Selective analysis of TCA metabolites from the untargeted metabolomics was performed in untreated control (C) and 10 mM metformin (M)-treated cells. (**A**) Heatmap showing alterations in TCA cycle metabolites in A2780, C200, and SKOV3ip cells. Scaled metabolite intensity graphs showing levels of (**B**) citrate, (**C**) succinate, (**D**) fumarate, and (**E**) malate. For all box-plot graphs, * *p* ≤ 0.05, NS = *p* non-significant, # *q* ≤ 0.05, ns = *q* non-significant when metformin treated compared to control.

The TCA cycle through oxidative phosphorylation is the major source of ATP production. Metformin, via a direct inhibition of complex 1 of the respiratory chain, modulates mitochondria function and thereby should affect TCA metabolites [39]. The alterations in the TCA pathway were again cell-line specific (Figure 3A). Except for significantly decreased malate and slightly elevated fumarate, metformin treatment did not result in robust changes in TCA cycle intermediates in A2780 cells. A potential cause of the decrease in malate may be the decreased conversion of fumarate to malate, which may further impact complex I of the mitochondrial respiratory chain and ATP production. In contrast, metformin treatment did have a significant effect on the TCA cycle intermediates in both C200 and SKOV3ip cells (Figure 3B–E). Metformin treatment resulted in significantly decreased (C200) and significantly increased (SKOV3ip) levels of all detected TCA cycle intermediates (citrate, succinate, fumarate, and malate) (Figure 3B–E). These changes suggest TCA activity is decreased in C200 and increased in SKOV3ip cells following metformin treatment. The major sources of acetyl CoA supplying the TCA cycle come from FA β-oxidation (FAO) and glycolysis, and it has been demonstrated that metformin can increase the activity of both pathways [40,41]. Thus, one potential cause of these cell-line specific differential effects of metformin on the TCA cycle may be the result of cell-line specific alterations of FAO (discussed below) and/or glycolysis (discussed above). Based on glycolysis metabolite alterations, a potential block in the late glycolytic intermediates occurs may reduce the feeding of the TCA cycle and the decreased intermediates in A2780 and C200 cells by metformin. In contrast, metformin treatment resulted in increased metabolism of glucose and a subsequent increase in pyruvate to lactate in SKOV3ip cells and may further explain why the TCA cycle intermediates were also elevated in SKOV3ip following metformin treatment. Thus, the inconsistent changes in the TCA cycle metabolites by metformin, even though metformin inhibits oxidative phosphorylation/ mitochondrial activity, further supports the idea that the altered metabolite profile of a particular metabolic pathway may not appropriately reflect the metabolic activity.

### 3.5. Fatty Acid β-Oxidation Was Increased by Metformin

**Figure 4:**
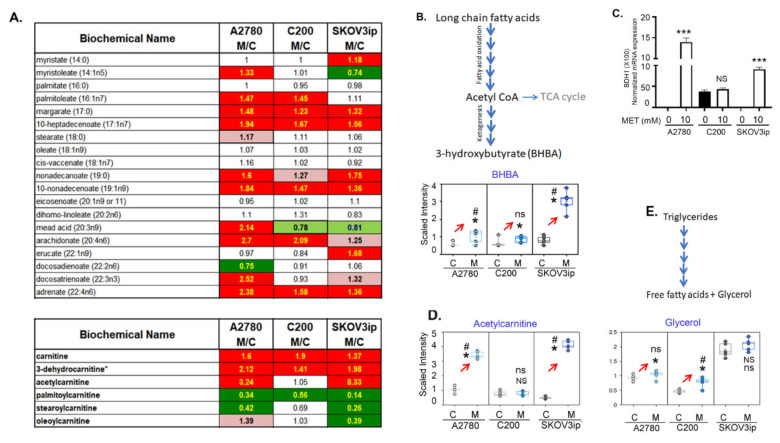
Increase in fatty acid metabolism by metformin. Selective analysis of lipid metabolism from the untargeted metabolomics was performed in untreated control (**C**) and 10 mM metformin (M)-treated cells. (**A**) Fold-change in mean metabolite intensity in fatty acids and carnitines in response to 10 mM of metformin treatment in A2780, C200, and SKOV3ip cells, relative to untreated cells. Dark green indicates a significant difference (FDR ≤ 0.05) between the groups; light green indicates differences approaching significance (0.05 ≤ FDR ≤ 0.10) between the groups shown; red indicates a significant difference (FDR ≤ 0.05) between the groups; and pink indicates differences approaching significance (0.05 ≤ FDR ≤ 0.1) between the groups. (**B**) Pathway depicting metabolism of ketone body BHBA (beta-hydroxybutyrate) and its log-transformed scaled intensity boxplot. (**C**) mRNA expression of BDH1 (beta-hydroxybutyrate dehydrogenase) transportation of long-chain fatty acids, scaled intensity showing levels (BHBA) in A2780, C200, and SKOV3ip after metformin treatment. *** *p* ≤ 0.001, NS = non-significant when metformin treated compared to untreated. (**D**) Scaled intensity levels of acetyl carnitine in A2780, C200, and SKOV3ip after metformin treatment. (**E**) Scaled intensity levels of glycerol in A2780, C200, and SKOV3ip after metformin treatment. For all box-plot graphs, * *p* ≤ 0.05, NS = *p* non-significant, # *q* ≤ 0.05, ns = *q* non-significant when metformin treated compared to control.

Metformin has been demonstrated to increase FAO [42]. Many of the long-chain FAs detected were significantly higher in the metformin-treated cells regardless of cell line (Figure 4A). Upon the conversion of FAs into an acyl-CoA, they must be conjugated to carnitine to be transported across the mitochondrial matrix for FAO. Carnitine and the carnitine degradation product, 3-dehydrocarnitine, were significantly elevated following metformin treatment in all three cell lines. In contrast, except for oleoylcarnitine (non-significantly elevated in A2780 and unchanged in C200 by metformin), the long-chain acyl-carnitines (palmitoylcarnitine, stearoylcarnitine, and oleoylcarnitine (SKOV3ip only)) were significantly lower in almost all cell lines following metformin treatment (Figure 4A). These changes in FAs and carnitines may be suggestive of increased FA lipolysis and high FAO. Supporting the increased FAO are the significantly increased levels of the ketone bodies 3-hydroxybutyrate (BHBA) and acetylcarnitine (Figure 4B,D), which are elevated when FAO is increased. Validating this, we observed the mRNA expression of 3 hydroxy butyrate dehydrogenase 1, and the enzyme that catalyzes acetoacetate to BHBA was increased in A2780 and SKOV3ip cells in response to metformin, whereas no significant difference was observed in C200 cells (Figure 4C). Thus, as previously shown, metformin increases FAO [27] leading to ketone bodies, most significantly in SKOV3ip cells, as seen by the greater elevation in BHBA and acetylcarnitine compared to A2780 and C200 following treatment. We also observed increased levels of glycerol, suggesting increased lipolysis in A2780 and C200 after metformin treatment. SKOV3ip cells have high basal levels of glycerol that did not change much after metformin treatment. Overall, metformin increases FAO across the three cell lines.

### 3.6. Alteration of Other Major Metabolic Pathways by Metformin

As seen in the heatmap (Figure S2), metformin treatment results in extensive metabolite changes affecting almost every metabolic pathway. Some of the notable alterations are described below.

#### 3.6.1. Metformin Inhibits the Pyrimidine Metabolic Pathway

Pyrimidines are precursors for the nucleic acids: cytosine, thymine, and uracil, the backbone of RNA and DNA. Most of the uridine associated pyrimidines (Uridine MonoPhosphate, UMP; Uridine DiPhosphate; UDP; Uridine TriPhosphate UTP, and uridine) were significantly decreased by metformin in all three cell lines (Figure S4B–E), with the exception being uracil (Figure S4F), which was non-significantly increased in A2780 and C200, and likely represents increased catabolism of pyrimidines. Although increased catabolism of pyrimidines can account for the decrease in these pyrimidines, another possible explanation may be a block in pyrimidine biosynthesis. In support of a block in pyrimidine biosynthesis following metformin treatment was the significant increase in the pyrimidine biosynthesis intermediate, orotate (Figure S4A), which may indicate a decreased pyrimidine biosynthesis due to decreased pentose phosphate pathway activity and/or ATP production following metformin treatment. The lower levels of pyrimidines may be a contributor or consequence of metformin’s antiproliferative effect.

#### 3.6.2. Metformin Decreases the Polyamine Biosynthesis

Polyamines, in particular spermidine and spermine, are known to regulate cancer cell proliferation [43]. Polyamines are produced via urea cycle metabolism associated conversion of ornithine to putrescine, which is then converted to spermidine and spermine. Polyamine levels, in particular spermidine, were significantly decreased by metformin, with the greatest decrease occurring in A2780 cells (Figure S5E). While C200 and SKOV3ip appear to have a decreased conversion of ornithine to putrescine following metformin treatment, A2780 cells increased the levels of putrescine, suggesting the effects of metformin on A2780 cells were occurring at the conversion of putrescine to spermidine (Figure S5A–D). Ornithine also contributes to proline production and the increase in proline in metformin treated cells may also contribute to the decrease in polyamines (Figure S5A). Regardless of the underlying mechanism, the lower levels of polyamines can contribute to metformin’s antiproliferative activity.

#### 3.6.3. Metformin Increases Essential Fatty Acids

All three cell lines showed increased levels of the essential FAs following metformin treatment (Figure S6). As these FAs are involved in the formation of inflammatory and anti-inflammatory lipids, the higher levels of the essential FAs may indicate decreased metabolism to inflammatory prostaglandins and 5-hydroxyeicosatetraenoic acid, thus supporting the role of metformin in regulating inflammation, as shown by others [44] and us [45].

### 3.7. Metformin Upregulates the Alpha-Linolenic and Linoleic Acid Metabolic Pathway

**Figure 5:**
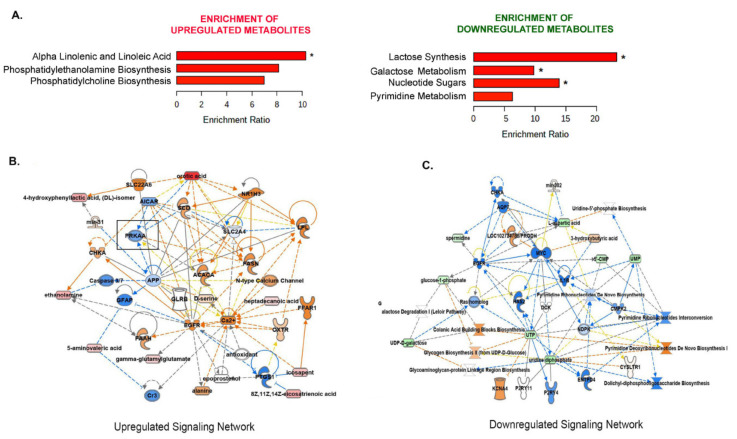
Enrichment analysis reveals commonly altered metabolic pathways in response to metformin. (**A**) The consistently upregulated and consistently downregulated metabolites across all the three cell lines (* FDR < 0.05) were explored through enrichment analysis (MetaboAnalyst, SMDB pathways) to highlight concerted alterations. The significantly enriched metabolic pathways (FDR < 0.05) are presented. Ingenuity pathway analysis (IPA)-constructed network relationships and directional predictions about the relationships between consistently upregulated (**B**) or downregulated metabolites (**C**). Dark blue lines = predicted inhibition, dark orange = predicted activation. The blue and orange arrows show that the relationship is as expected for this prediction. Yellow arrows indicate that relation is not as expected. The observed metabolite fold change is in the shades of pink (up) and green (down) ovals.

The metabolic component of metformin’s antiproliferative effect must be consistent in metformin sensitive cell lines, meaning affected metabolites must increase or decrease in all three cell lines and affect the same biological pathways. Thus, we performed a metabolite set enrichment analysis using the 60 metabolites that changed across the three cell lines (Figure S2D) to identify biologically meaningful patterns that are significantly enriched in the quantitative metabolomics profile [46]. Of these, 12 are down consistently and 22 are up consistently across the cell lines, while other metabolites are increased in some and down in other cells. A combined analysis of the 60 significantly (FDR ≤ 0.05) altered metabolites common (both directions) in all three cell lines revealed alpha-linolenic (ALA) and linoleic acid (LA) as the most commonly enriched metabolic pathway, while lactate synthesis, galactose metabolism, and nucleotide sugars were the most significant downregulated pathways (Figure 5A). The ALA and LA are essential FAs and belong to the class of polyunsaturated fatty acids (PUFAs). These essential FAs are precursors of lipids having diverse physiological functions, including modulation of inflammation and immune response, regulation of proliferation, and transcriptional regulation [47].

To determine the biological and regulatory context of the enriched metabolic pathways, an IPA of the enriched metabolites was performed to understand the signaling networks being modulated by these metabolites. Amino acid metabolism, molecular transport, and small molecular biochemistry presented as the most significantly affected signaling network, which may suggest the accumulation of amino acids, due to a block in the protein synthesis biochemistry as expected and observed in response to metformin [48]. The second most upregulated pathway was the lipid metabolism, small molecule biochemistry, molecule transport, where AMPK, the downstream target of metformin, emerged as a prominent signaling hub (Figure 5B). Cellular growth, proliferation, organismal growth, and cancer was the most significant downregulated signaling network in response to metformin, which was reflective of decreased metabolic pathways of pyrimidine biosynthesis and polyamine synthesis (Figure 5C). This also agrees with metformin’s widely established antiproliferative effects on cancer cells. Interestingly, the downregulated metabolites in the network belong to those involved in the nucleotide biosynthesis, which may translate to blocks in RNA transcription and protein biosynthesis, known to be affected by AMPK and metformin [17,49].

**Figure 6:**
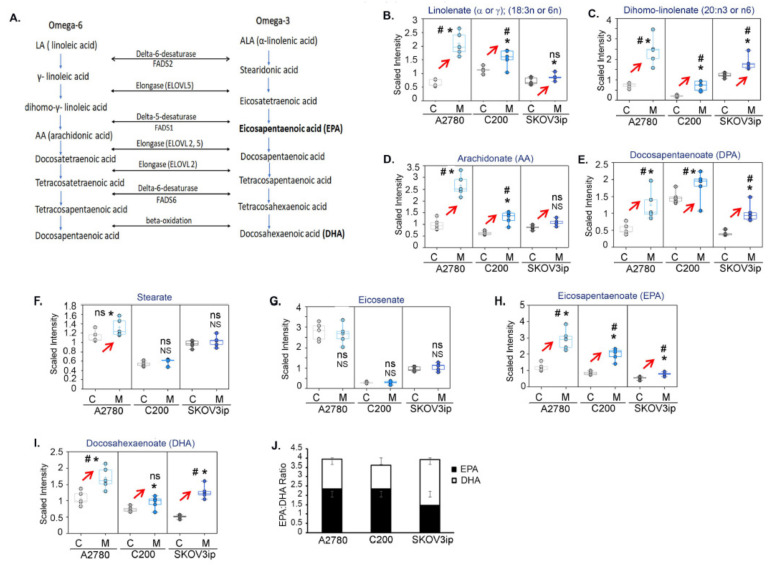
Metformin enhances DHA and EPA metabolism. (**A**) Schematic briefly depicting the omega-6 (LA, linoleic acid) and omega-3 fatty (ALA, alpha-linolenic acid) acid metabolism leading to generation of downstream lipid mediators. Several metabolite intermediates that were increased in most cells by metformin included (**B**) linolenate, (**C**) di-homo linolenate, (**D**) arachidonate (AA), (**E**) docosapentaenoate (DPA), (**F**) Stearate, (**G**) eicosenate, (**H**) eicosapentaenoate (EPA), and (**I**) docosahexaenoate (DHA). (**J**) Ratio of the level of EPA and DHA in A2780, C200, and SKOV3ip. For all box-plot graphs, * *p* ≤ 0.05, NS = *p* non-significant, # *q*≤ 0.05, ns = *q* non-significant when metformin treated compared to control.

Due to the known multifaceted roles played by PUFAs as important structural components of the cell membrane, energy metabolism, and precursors to bioactive lipid mediators, we further investigated the omega-6 and omega-3 FA pathway downstream of ALA and LA (Figure 6A). Long-chain omega-3 PUFAs, EPA, and DHA are known to exert anti-inflammatory and anticancer effects [47]. Many of the intermediates downstream of omega-3 ALA and omega-6 LA (Figure 6B–I) were increased in all three cell lines in response to metformin. Both EPA and DHA were increased in all three cell lines, with a higher EPA:DHA ratio in A2780 and C200 compared to SKOV3ip cells (Figure 6J), indicating that the levels of EPA are significantly higher compared to DHA, at least in the A2780 and C200 cell lines.

Thus, the ability of metformin to promote the production of omega-3 metabolites DHA and EPA may partially be responsible for mediating its antitumor effects.

### 3.8. EPA Is More Effective than DHA in Restricting Ovarian Tumor Growth and Improving Survival

Many studies have detailed the ability of omega-3 FAs, or EPA or DHA, to modulate cancer cells by reducing viability [50,51], by cell cycle arrest [52], or by inducing apoptosis [53] in various cancer models. We tested the effect of DHA and EPA on these parameters in the three cell lines and included an additional cell line CaOV3, identified as a true representative of high-grade serous ovarian cancer [54]. Both DHA and EPA inhibited the viability of all cell lines in a dose-dependent manner to a variable extent (Figure S7A,B). EPA induced a G0/G1 arrest with a decrease in cell fraction in the ensuing G2 and S phase in CaOV3 and SKOV3ip cell lines, compared to DHA, whereas no cell cycle arrest was seen in A2780 and C200 cell lines (Figure S7C–E). Hoechst staining showed EPA induced significant apoptosis, when compared to DHA, in all cell lines, except in C200 cells, where DHA treatment resulted in more apoptosis compared to EPA (Figure S7F). Thus, as observed by others, DHA and EPA inhibited ovarian cancer cell growth by different approaches in a cell line specific manner.

**Figure 7:**
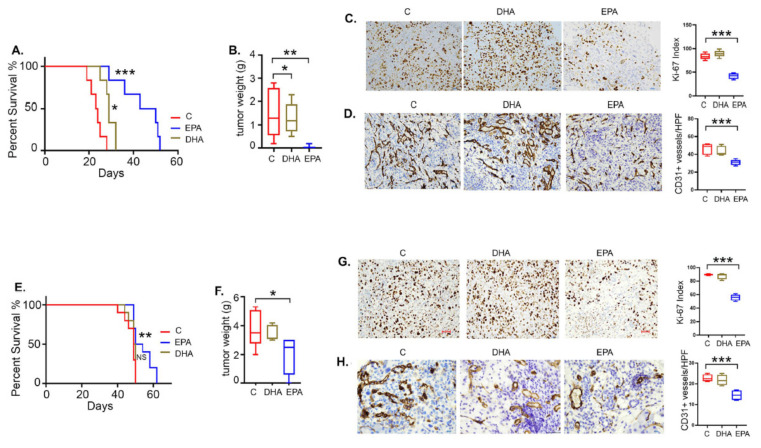
EPA and DHA inhibit ovarian cancer growth and improve survival. Mice injected intraperitoneally with (**A**) CaOV3 and (**E**) SKOV3ip were treated with EPA or DHA and observed for survival (*n* = 12), as described in the Materials and Methods section. Bar graph showing average tumor weights isolated from the (**B**) CaOV3 and (**F**) SKOV3ip mouse models (*n* = 10 mice per group). Tumor sections from both groups were subjected to immunohistochemistry (IHC) for proliferation and neovascularization markers. Representative IHC images (200×) showing Ki67 stain in CaOV3 (**C**) and SKOV3ip (**G**) tumors. Box-plots show the Ki-index calculated from *n* = 5 images per group at high-power field (HPF). Representative IHC images (200×) showing CD31 stain in CaOV3 (**D**) and SKOV3ip (**H**) tumors. Box-plots show the number of positive vessels per field calculated from *n* = 5 images per group at HPF. * *p* ≤ 0.05, ** *p* ≤ 0.01, and *** *p* ≤ 0.001, respective treated group compared to vehicle-treated control.

For in vivo validation, we tested the efficacy of DHA and EPA in the SKOV3ip and CaOV3 xenograft models. The DHA and EPA treatments were given by gavage (100 mg/kg body weight), while the control mice received equal amounts of corn oil as vehicle. No significant difference was observed in the weight of the omega-3 FA-fed or vehicle-fed mice in both cell line models [48]. Interestingly, EPA was more effective compared to DHA in both models, while its efficacy was higher in CaOV3 compared to the SKOV3ip model. In the CaOV3 model, EPA increased the median survival by more than 20 days (median survival 46.5 days) compared to the vehicle control (median survival 23.5 days), whereas DHA improved the survival to a lesser extent (median survival 29 days) (Figure 7A). A similar although less potent effect was observed in the SKOV3ip model, where EPA increased the median survival (56 days) by 7 days compared to the vehicle control (median survival 49 days), whereas improvement with DHA was not significant (49.5 days) (Figure 7E). The improved survival was reflected in the significantly decreased excised tumor weights in EPA-treated mice in contrast to DHA-treated mice in both the CaOV3 and SKOV3ip models (Figure 7B,F). The smaller tumor sizes in EPA-treated mice were further validated by decreased proliferation, as assessed by the Ki-67 index, compared to control and DHA mice in both the CaOV3 and SKOV3ip models (Figure 7C,G). The bar graphs represent quantification of the Ki-67 index calculated as a ratio of Ki-67 positive cells to total tumor cells [35]. Decreased CD31 staining in EPA-treated tumors compared to the vehicle control and DHA-treated mice, indicated decreased angiogenesis (Figure 7D,H). The bar graphs represent quantification of CD31 positive cells per high power field (HPF).

Taken together, exogenously provided EPA is more effective than DHA in reducing ovarian tumor growth, although this may be associated with the cancer cell type and metabolic phenotype.

### 3.9. EPA Induces Apoptotic Cell Death in Ovarian Tumors

**Figure 8:**
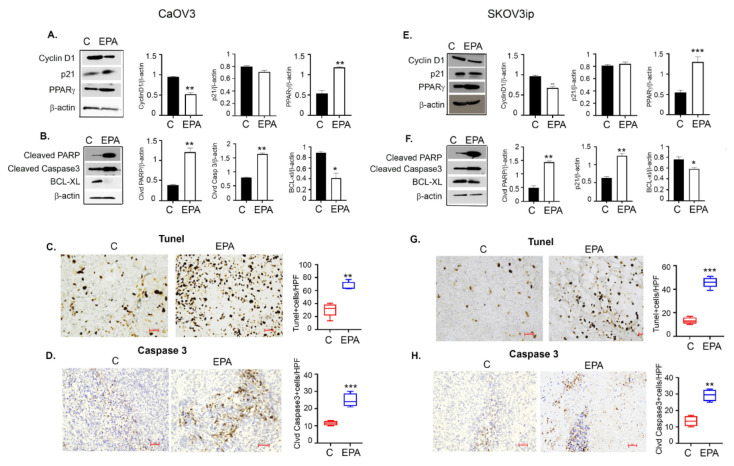
EPA treatment induces antiproliferative and apoptotic cell death in ovarian tumors. Pooled tumor tissue (*n* = 4) was used to isolate protein and immunoblotted for various markers including cell cycle proteins cyclin D1 and p21, and proliferation promoter PPARγ in (**A**) CaOV3 and (**E**) SKOV3ip tumors treated with EPA or vehicle. Bar graph represents normalized densitometric expression, *n* = 2. Uncropped Western blot images are shown in Figure S9. Immunoblots showing expression patterns of apoptotic markers cleaved PARP and cleaved caspase 3 in (**B**) CaOV3 and (**F**) SKOV3ip tumors treated with EPA or vehicle. Bar graph represents normalized densitometric expression, *n* = 2. Uncropped Western blot images are shown in Figure S9. Representative IHC images (200×) showing TUNEL stain in CaOV3 (**C**) and SKOV3ip (**G**) tumors. Box-plots show the number of positive cells per field calculated from *n* = 5 images per group at HPF. Representative IHC images showing cleaved caspase 3 stain in CaOV3 (**D**) and SKOV3ip (**H**) tumors. Box-plots show the number of positive cells per field calculated from *n* = 5 images per group at HPF. * *p* ≤ 0.05, ** *p* ≤ 0.01, and *** *p* ≤ 0.001, EPA-treated group compared to vehicle-treated control.

Since we observed EPA to be more effective, hereafter we focused on mechanisms associated with EPA. As observed in vitro, EPA treatment decreased the expression of the proliferation promoting cell cycle regulatory protein cyclin D1 [55] in CaOV3 and SKOV3ip tumors (Figure 8A,E), while no effect was seen in the expression of the cyclin dependent kinase inhibitor p21 [56]. Omega-3 FAs are natural ligands for PPAR, where PPARγ has been shown to have antiproliferative and antigrowth effects [57]. EPA-treated CaOV3 and SKOV3ip tumors showed increased PPARγ expression compared to the control, indicating its role in EPA-mediated ovarian cancer cell inhibition (Figure 8A,E). The bar graphs represent the densitometry from a minimum of two Western blots run from pooled tumor samples for each antibody.

Similarly, EPA treated tumors showed increased expression of apoptotic markers including cleaved caspase 3 and cleaved PARP compared to the control in both models (Figure 8B,F). Expression of BCL-xl, an anti-apoptotic member of the BCL-2 family, was decreased in EPA-treated tumors compared to control tumors of both models (Figure 8B,F). Increased cleaved caspase 3 induced by EPA was also confirmed by immunohistochemistry in CaOV3 (Figure 8D) and SKOV3ip (Figure 8H) tumor sections. Apoptosis was further confirmed by performing the TUNEL staining, indicating the final DNA fragmentation step of the apoptotic pathway [58], which was increased in EPA-treated CaOV3 and SKOV3ip tumors compared to the control (Figure 8C,G). The bar graph represents the quantification of the percentage of positive (brown) cells per HPF.

Overall, these data demonstrate that EPA can induce cell cycle arrest and apoptosis in preclinical ovarian cancer models.

### 3.10. EPA Treatments Lower the Inflammatory Milieu in Ovarian Tumors and Its Microenvironment

**Figure 9:**
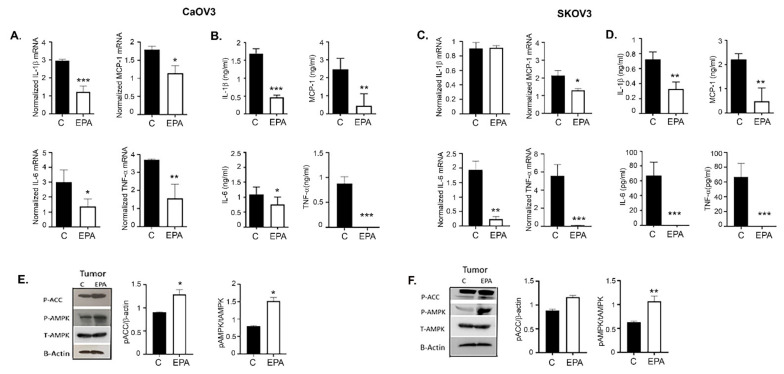
EPA lowers the inflammatory milieu in ovarian tumors and its microenvironment. Pooled mRNA from tumor tissues (*n* = 3) was assayed for inflammatory of interleukin (IL)-1β, MCP-1, IL-6, and TNF-α in (**A**) CaOV3 and (**C**) SKOV3ip mice treated with EPA or vehicle. Protein levels of IL-1β, MCP-1, IL-6, and TNF-α in plasma (*n* = 3) isolated from (**B**) CaOV3 and (**D**) SKOV3ip mice treated with EPA or vehicle. (**E**,**F**) Representative immunoblots showing phosphorylated (p)-ACC, AMPK and total (T) AMPK and β-actin expression from pooled tumors (*n* = 3) from vehicle (C) or EPA treated mice. Bar plots show normalized densitometric analysis from two individual blots. Uncropped Western blot images are shown in Figure S9. * *p* ≤ 0.05, ** *p* ≤ 0.01, and *** *p* ≤ 0.001, EPA-treated group compared to vehicle-treated control.

Inflammation is an important component associated with ovarian cancer and its tumor microenvironment [59]. Omega-3 FAs are intimately involved in regulating the inflammatory processes and modulating cell function [60]. EPA treatment inhibited the mRNA expression of inflammatory cytokines including IL-1β, IL-6, MCP-1, and TNF-α in both the CaOV3 (Figure 9A) and SKOV3ip tumors (Figure 9C), which was validated by similar decreased levels of these inflammatory cytokines in the serum from mice of both tumor models (Figure 9B,D).

We previously reported that metformin inhibits ovarian tumor growth by inducing AMPK activation [45], and one of the mechanisms by which metformin limits inflammation is by activation of AMPK [44]. Omega-3 FAs have been shown to activate AMPK in muscle [61] and cancer cells [62]. Tumors from EPA-treated mice of both CaOV3 and SKOV3ip models showed increased expression of pAMPK and its surrogate marker, pACC (acetyl Co-carboxylase A), compared to the control (Figure 9E,F). Bar graphs represent the Western blot densitometry of a minimum of two blots.

Overall, these data suggest that EPA can activate AMPK, which may mediate EPA-mediated inhibition of ovarian tumor growth, and indicates a multilayer crosstalk between AMPK omega-3 FAs and modulation of metabolism that eventually regulates cell growth.

### 3.11. Metformin and EPA Induced the Downstream Anti-Inflammatory Pathway Mediators

Omega-3 FAs are metabolized to produce the downstream lipid mediators called specialized pro-resolving lipid mediators (SPMs) via the action of various LOX and COX enzymes. DHA produces the family of protectins, maresins, and Resolvin D (RvD 1–6), whereas EPA produces the Resolvin E family (RvE1–3) of SPMs [63]. SPMs have been known to possess anti-inflammatory and immunomodulatory abilities [64], with recent evidence showing their roles in stem cells and cancer [65].

**Figure 10:**
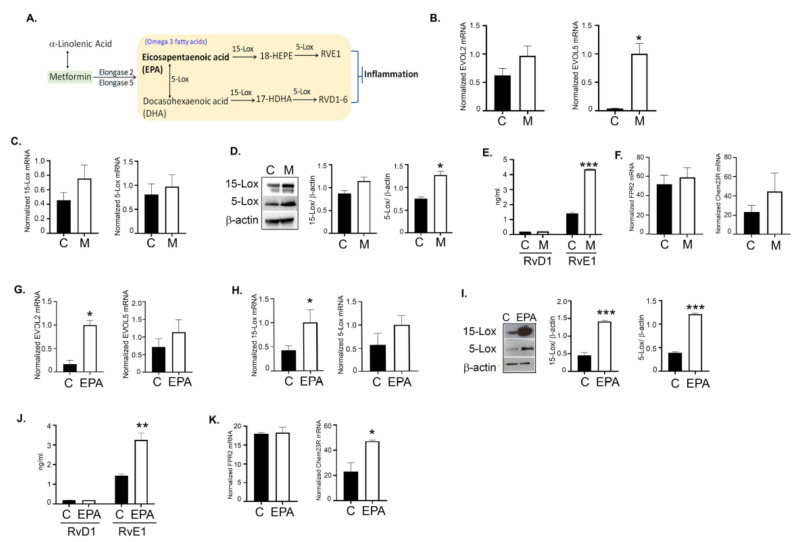
Metformin and EPA promote formation of specialized pro-resolving mediators. (**A**) A simplified schematic of the source of SPMs from EPA and DHA. (**B**) mRNA expression of ELOVL2 and ELOVL 5 in pooled CaOV3 tumors (*n* = 3) treated with metformin or vehicle control. (**C**) mRNA expression and (**D**) protein expression of 5-LOX and 15-LOX in pooled CaOV3 tumors (*n* = 3) treated with metformin or vehicle control. Immunoblots are representative of two independently run blots. Bar graphs are normalized densitometric measurements (*n* = 2). Uncropped Western blot images are shown in Figure S9. (**E**) RvD1 and RvE1 protein levels measured in plasma isolated from CaOV3 tumors from mice treated or untreated with metformin (*n* = 4). (**F**) mRNA expression of FPR2 and Chem23R in CaOV3 tumors (*n* = 3) treated with metformin or control (*n* = 3). (**G**) mRNA expression of ELOVL2 and ELOVL 5 in pooled CaOV3 tumors (*n* = 3) treated with EPA or vehicle control. (**H**) mRNA expression and (**I**) protein expression of 5-LOX and 15-LOX in pooled CaOV3 tumors (*n* = 3) treated with EPA or vehicle control. Immunoblots are representative of two independently run blots. Bar graphs are normalized densitometric measurements (*n* = 2). Uncropped Western blot images are shown in Figure S9. (**J**) RvD1 and RvE1 protein levels measured in plasma isolated from CaOV3 tumors treated with EPA or vehicle control (*n* = 4). (**K**) mRNA expression of FPR2 and Chem23R in CaOV3 tumors (*n* = 3) treated with EPA or vehicle control (*n* = 3). * *p* ≤ 0.05, ** *p* ≤ 0.01, and *** *p* ≤ 0.001, respective treated group compared to control.

To investigate if metformin promoted omega-3 FA metabolism to produce SPMs, we induced CaOV3 tumors followed by metformin (200 mg/kg body weight) treatment as before, and examined the expression of various enzymes involved in the metabolism of omega-3 FAs to SPMs (Figure 10A). Metformin-treated tumors showed increased expression of the enzymes involved in DHA and EPA metabolism from ALA, specifically ELOVL2 (elongase 2) and ELOVL 5 (elongase 5) from ALA (Figure 10B). EPA is metabolized by 15-LOX to 18-hydroxy eicosapentaenoic acid and further to the RvE series of SPMs by 5-LOX. In parallel, EPA is also metabolized to DHA by 5-LOX (Figure 10A). DHA is further metabolized in the presence of 15-LOX/5-LOX to the RvD family and maresin in the presence of 12-LOX. Metformin treatment showed a trend towards increased expression of 5-LOX and 15-LOX in tumors compared to the control at mRNA and protein levels (Figure 10C,D). Strikingly, metformin treatment significantly increased RvE1 levels in plasma compared to the control, whereas there was no significant difference in RvD1 levels (Figure 10E). To be positive that ovarian tumors express the receptors to mediate the SPM signaling, we tested for the expression of FPR2 (RvD1 receptor) [66] and Chem23R (RvE1 receptor) [67], which were both expressed in the CaOV3 tumors. Further, metformin treatment slightly increased the expression of Chem23R, whereas FPR2 expression was unchanged (Figure 10F). Together, this suggests that metformin promotes EPA metabolism, resulting in the production of the RvE1 series of SPMs, rather than the RvD1 series. EPA-treated tumors also exhibited a similar increase or a trend towards increase in the expression of ELOV2, ELOV5 (Figure 10G), 15-LOX, and 5-LOX (Figure 10H–I), followed by increases synthesis of RvE1 (Figure 10J). Like metformin, EPA did not affect FPR2 expression, whereas an increase in ChemR23 was observed compared to the control (Figure 10K). A similar trend in enzyme changes following EPA treatment was also observed in the SKOV3ip model (Figure S8E). Although we observed the same trend in SKOV3ip tumors, significance was not achieved in the levels of RvE1 (Figure S8C). This may explain the reduced effect of EPA in SKOV3ip tumors compared to CaOV3 tumors (Figure 7).

Overall, these data strongly indicate that metformin promotes the metabolism of EPA into its downstream SPM mediators (RvE1), and, as expected, EPA treatments increase the production of RvE1 SPM, which may be responsible for the decreased inflammatory tumor environment contributing to decreased tumor burden.

## 4. Discussion

Metformin, which is the most widely prescribed drug for type 2 diabetes, and has a well-established safety profile and low cost, is deemed to be an “essential medicine” by the World Health Organization [68], and is being actively pursued for repurposing for the treatment and prevention of various cancers, including ovarian cancer [69]. In ovarian cancer, we and others have demonstrated the anti-tumor effects of metformin via multiple mechanisms in various preclinical models [2,3,4,6]. We and others have reported retrospective patient studies supporting the anticancer benefit of metformin in patients, with most performed in type 2 diabetes patients [7,8,9,70]. These studies have led to active testing of metformin in clinical trials. In a recently reported nonrandomized phase II clinical trial (NCT01579812), Brown et al. demonstrated metformin was well tolerated in nondiabetic stage II, III, and IV ovarian cancer patients and improved overall survival, with metformin-treated tumors showing a significant reduction in the cancer stem cell population and increased sensitivity to cisplatin. This study strongly supports the use of metformin in phase III clinical trials for adjuvant treatment of epithelial ovarian cancer [71]. In contrast, a small pilot randomized clinical trial of metformin in combination with first-line chemotherapy did not show a meaningful effect on progression-free survival, although a positive trend was observed and the negative modulation of the pro-tumor IGF-1 signaling axis was observed [72]. Other clinical trials include a phase 1b (NCT02312661) and a randomized phase II trial (NCT02122185) that are studying the efficacy of metformin when combined with chemotherapy drugs in patients with advanced ovarian cancer. Thus, repurposing metformin for gynecologic cancers is an attractive option and needs to be further investigated.

Although an extensive body of research has defined the anticancer mechanisms of metformin, these are widely debated and span multiple pathways affecting cellular, molecular, and physiological processes [16,17,73,74]. An important property of metformin is its interference with mitochondrial biology due to inhibition of the mitochondrial complex 1 [15,75], which creates an energy imbalance leading to activation of AMPK [13], although metformin’s ability to activate AMPK independent of energy imbalance has also being described [22,76]. Mitochondria are also the metabolic powerhouse that controls the metabolism of various sugars, amino acids, and fatty acids to maintain the bioenergetic status of a cell and feed the various biosynthetic pathways for biomass production. Thus, via its effects on mitochondria and pleiotropic effects on oncogenic signaling, metformin can alter the cellular metabolism. Although various studies have described the functional changes in the bioenergetics of glycolysis, TCA, and/or lipid oxidation respiration in response to metformin in various cancer types [27,28,77,78], few studies have addressed the global metabolic consequences of metformin in cancer. Corominas-Faja et al. performed metabolic fingerprinting in breast cancer cell lines and showed metformin impaired one-carbon metabolism, affecting the purine/pyrimidine de novo synthesis and glutathione, and acting in a similar manner as antifolates [49]. Schuler et al. performed untargeted metabolomics in a preoperative window study in obese endometrial cancer patients and found metformin induced a shift in lipid and glycogen metabolism that was more pronounced in the serum and tumors of responders versus non-responders [27]. Recently, Liu et al. demonstrated extensive and heterogenous cellular metabolic reprogramming in the tumors of metformin, using ovarian cancer patients and experimental mouse models [31]. They identified mitochondrial associated metabolic alterations in the TCA cycle and defects in FAO as key events and reported the response to metformin to be dependent on the flexibility of substrate utilization by the mitochondria of the cancer cell.

In our study, we aimed to identify the common metabolic alterations that can be indicators and mediators of metformin’s antitumor effects. Untargeted metabolic profiling of isogenic chemosensitive and resistant cell lines A2780 and C200, and the highly aggressive and mutated cell line SKOV3ip, showed the extensive effects of metformin in changing the metabolism of almost every metabolic pathway. Due to its effect on the mitochondrial complex 1, we anticipated consistent changes in the energy metabolism of the cells. Although metformin had an immediate and continued effect on the functional energetics, as it consistently inhibited mitochondrial oxidation and induced glycolysis as expected across the cell lines, the metabolites of the glycolysis and TCA cycle were inconsistently changed across the three cell lines. These inconsistent metabolite levels may be due to the inherent metabolic adaptations of the individual cell line or the specific oncogenic gene mutations. Previously, we showed the chemoresistant and aggressive ovarian cancer cell lines were metabolically flexible, having greater preference for mitochondrial oxidation [34], which may reflect the upregulation of TCA over glycolysis or an upregulation of both the metabolic pathways. The pronounced changes observed in SKOV3ip, such as the increased glycolysis intermediates, may be due to other adaptations, such as the increased expression of Glut 1, enabling increased glucose uptake. In addition, SKOV3ip cells carry various oncogenic mutations of PI3K and ARID1A pathways, known to modulate the nutritional sensor pathway mTOR and activate various other metabolic genes [79]. We also observed downregulation of the pyrimidine biosynthesis pathway, which has been reported by others in breast and hepatocarcinoma cells [49]. Inhibition of polyamine biosynthesis, associated with cell growth and proliferation, was also consistent across the cell lines. The recently improved understanding of cancer metabolism has led to re-recognition of targeting polyamine metabolism in cancer cells and the antitumor immune response [43]. One of the most consistent changes was observed in the FA metabolism, with FAO being upregulated in all the cell lines, as reflected by increases in FAs, carnitines, ketone bodies, and glycerol. Thus, our study shows metformin affects mitochondrial regulation of metabolic processes with consistent changes in lipid catabolism, as in other studies.

Although myriad and heterogenous changes were observed across the cell lines, enrichment analysis revealed that ALA and LA metabolism was the common significantly upregulated pathway. ALA and LA are the essential omega-3 and omega-6 FAs, respectively. Both omega-6 and omega-3 FAs perform vital functions as part of structural components of cell membranes, energy sources, and precursors to various lipid mediators that control various cellular functions. Omega-3 FAs are known to exert antiproliferative and anti-inflammatory functions in various cancers and other diseases. Both ALA and LA metabolize into longer PUFAs, including arachidonic acid (AA), EPA, and DHA through a series of desaturation and elongation reactions carried out by the shared elongase and desaturase enzymes [80,81]. Metformin treatment resulted in increased levels of various intermediates of the ALA and LA metabolism and expression of elongases, indicating that metformin favors the metabolism of omega-3 FAs. Mounting evidence from experimental studies strongly indicate that omega-3 PUFAs, particularly EPA and DHA, have antineoplastic effects alone, and more in combination with various chemotherapy drugs, in multiple cancer types [47]. These omega-3 FAs have been extensively investigated in numerous clinical trials for various diseases, including cancer and cancer-associated cachexia [47]. In ovarian cancer, omega-3 FA supplementation or diets rich in omega-3 FAs have been shown to inhibit growth or cause apoptosis in vitro by various mechanisms ranging from suppression of proliferative signals, oncogenes, and production of inflammation-resolving mediators [50,82,83].

Under our experimental conditions, we found EPA to be more effective than DHA in improving overall survival, reducing tumor burden, reducing proliferation, suppressing inflammation, and inducing apoptosis, although the effect was more pronounced in CaOV3 compared to SKOV3ip model. EPA treatments also resulted in increased expression of PPARγ as expected [57], and also induced activation of AMPK in livers and tumors. A few reports have earlier reported omega-3 FAs to induce AMPK in different cell types [61,84,85]. AMPK activation by metformin and other means has been shown to reduce inflammation in various disease models including cancer models [86]. This suggests a loop or synergy between the action of metformin and EPA that decreases inflammation and inhibits tumor growth by activating AMPK and other common mediators such as PPARγ. In support of our results, EPA has been shown to inhibit the growth of ovarian cancer cell line SKOV3 with an immunomodulatory role [87,88], and Zhao et al. showed that EPA suppresses ES2 ovarian clear cell carcinoma cells by inducing apoptosis [89]. Tanaka et al. reported that DHA and EPA induced ovarian cancer cell death through ROS and p38 activation [51]. Studies in other cancers have shown that EPA can induce autophagy and apoptosis in pancreatic cancer [53] and improve sensitization to various chemotherapy drugs in esophageal cancer cells [90]. In contrast, Wan et al. showed DHA to be more effective in suppressing the growth of TOV21G ovarian cancer cells partly mediated by PPARγ and p53 [91]. This discrepancy may be due to the differences in the cell lines used between the studies. Overall, DHA has been investigated more in ovarian cancer, with many reports showing DHA inhibiting ovarian cancer in vitro and in vivo by modulating the oncogenic signaling of NFkB and MAPK pathways, PPARγ activation, ROS activation, and potentiating chemotherapy [51,92,93]. However, none of these reports tested EPA by itself or in combination with DHA. Various studies have reported EPA as a better nutraceutical in amelioration of cancer cachexia [94,95].

Although metformin via its effect on various metabolic enzymes is known to regulate lipid metabolism, which can affect the FA profile of the tumors and have a direct impact on the membrane structure and cellular function [96,97], its direct effect on omega-3 FA metabolism with respect to DHA/EPA is not well studied. In a mouse model of breast cancer, Checkley et al. showed metformin reduced the n-6/n-3 FA ratio, which was mainly derived by reduced AA and increased DHA and LA, and found the tumor response to metformin was partially dependent on the reduction in AA [98]. Our studies show that metformin appears to favor the metabolism of EPA in the tumors, reflected by increased EPA levels and increased expression of enzymes involved in ALA metabolism, although this may be cell-type specific, as SKOV3ip cell lines showed more DHA levels compared to A2780 and C200. Although cancer studies have focused more on the combination of omega-3 FAs or on DHA, recent evidence from human studies indicates that there are more benefits from EPA compared to DHA [99,100]. A perspective on these studies suggests that the better efficacy of EPA may be due to the higher biological activity of an EPA-associated dose-dependent decrease in inflammation markers and the possibility that DHA may antagonize some effects of EPA [101]. Although DHA and EPA combinations have been studied together, EPA and DHA have distinct tissue distributions, including distinct sub-membrane localization and downstream effects on lipids, oxidation, and inflammation [102]. Thus, a comprehensive study of the effects of the role and biology of individual omega-3 FAs is warranted to better understand the applicability of these in cancer.

DHA and EPA are metabolized in the presence of LOX and cyclooxygenases to produce SPMs, such as resolvins, protectins, and maresins [63]. Mammalian LOX consists of 5-LOX, 12-LOX, and 15-LOX. Both DHA and EPA share 15-LOX and 5-LOX and are converted to their respective SPM families. DHA metabolism results in the SPM families of the RvD series, protectins, and maresins. In contrast, EPA uses the same LOX enzymes to produce the RvE series of SPMs [103,104]. In parallel, EPA is metabolized to DHA, and DHA can be converted to EPA [105]. Although the cross-sharing and inter-conversion makes it difficult to identify the exact pathway, metformin-treated mice showed increased expression of 15-LOX and 5-LOX, and, most interestingly, increased levels of RvE1 in the serum and increased expression of its receptor, ChemR23 [106], on tumor cells, in contrast to DHA-derived RvD1 and its receptor FPR2 [66]. EPA-treated tumors also showed a similar profile of increased ALA metabolism and production of RvE1. This is a very interesting observation, in which we see metformin promotes the synthesis of EPA-derived SPMs. Although various LOX enzymes have been described to have pro-tumor or antitumor roles, and have been targeted in various cancers [107], the role of SPMs has been studied in inflammation-related cancer, such as colorectal cancer [108] and gastric cancer [109], and is an emerging field in other cancers [64]. Using various preclinical cancer models, including an ovarian cancer model, a recent study by Sulciner et al. elegantly described the potential of resolvins (E1, D1, and D2) to suppress tumor growth and recurrence by limiting the inflammatory damage caused by apoptosis in response to cytotoxic chemotherapy, and by polarizing the proinflammatory macrophages towards phagocytic anti-inflammatory macrophages [110]. Thus, the omega-3 FA-derived SPMs may have the potential to be utilized as a part of therapeutic approaches against ovarian cancer.

## 5. Conclusions

Collectively, our study identified that metformin treatment results in enrichment of omega-3 metabolism; specifically, it enhances EPA metabolism and its downstream SPMs, which were associated with improved targeting of ovarian cancer cells and increased overall survival. Thus, clinical studies may need to focus on the status of lipid metabolism of ovarian cancer while designing patient studies, and to rethink the application of metformin in terms of a precision medicine approach. Although in-depth studies are required to define the role of SPMs as a therapeutic, our study supports their beneficial role in ovarian cancer.

## Data Availability

All data generated or analyzed during this study are included in this published article (and its Supplementary Material).

## Supplementary Materials

The following supporting information can be downloaded at: https://www.mdpi.com/article/10.3390/cancers14061504/s1, Figure S1: Metformin inhibits ovarian cancer cell proliferation. Figure S2: Global metabolomics analysis of ovarian cancer cells after metformin treatment. Figure S3: Effect of metformin on glycolysis cycle intermediates is cell line specific. Figure S4: Effect of metformin on pyrimidine metabolic pathway. Figure S5: Effect of metformin on polyamine metabolic pathway. Figure S6: Effect of metformin on essential FA metabolism. Figure S7: EPA and DHA inhibit growth and induce apoptosis in ovarian cancer cell lines vitro. Figure S8: EPA has no effect on formation of resolving E1 in SKOV3ip model. Figure S9: Uncropped Western blot images. Table S1: List and sequence of the primers used in the study. Table S2: List of the antibodies with catalogue number and dilution used. Table S3: List of the ELISA kits with catalogue number used in the study. Table S4: List of the antibodies used in immunohistochemistry with catalogue number.
