# Supplementary material for: Divergent Metabolic Effects of Metformin Merge to Enhance Eicosapentaenoic Acid Metabolism and Inhibit Ovarian Cancer In Vivo
Source: Cancers (Basel). 2022 Mar 15;14(6):1504. doi: 10.3390/cancers14061504 (PMC8946838; doi:10.3390/cancers14061504)
Supplement: Supplementary file 1 [file cancers-14-01504-s001.zip › cancers-1580739-supplementary.pdf]

Figure S1: Metformin inhibits ovarian cancer cell proliferation.

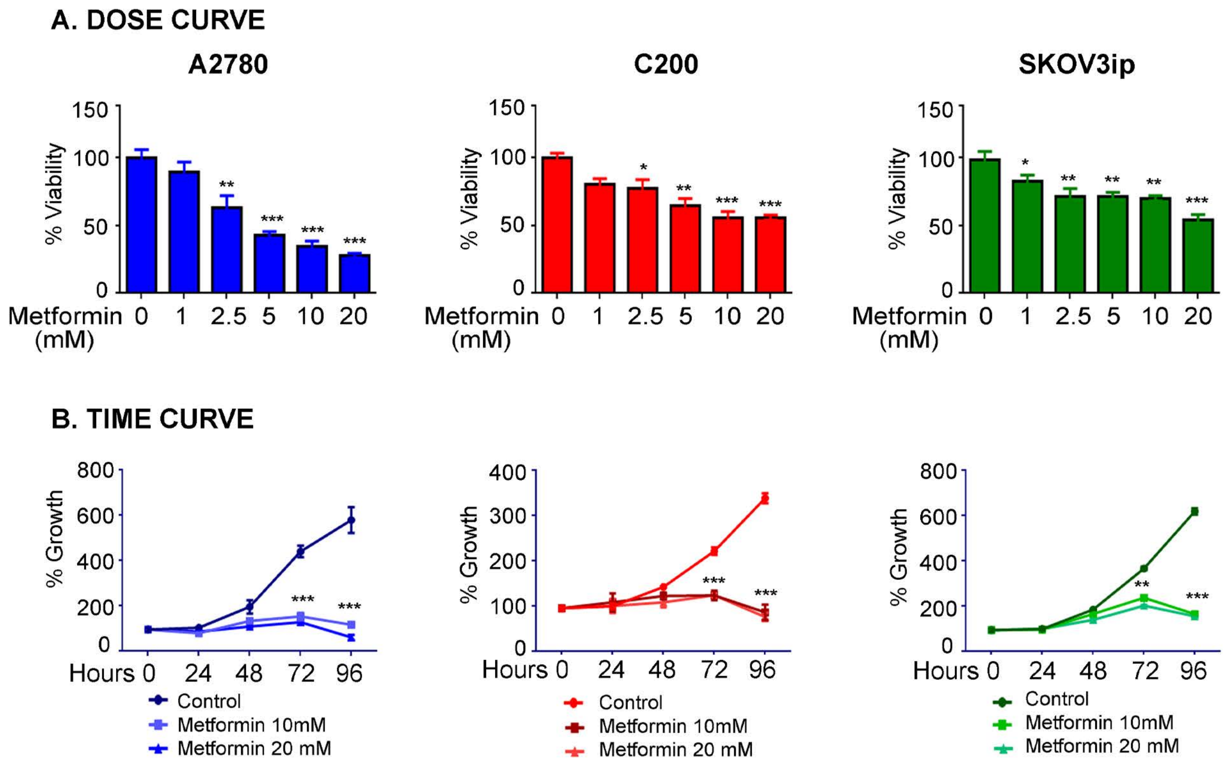

Figure S2: Global metabolomics analysis after metformin treatment.

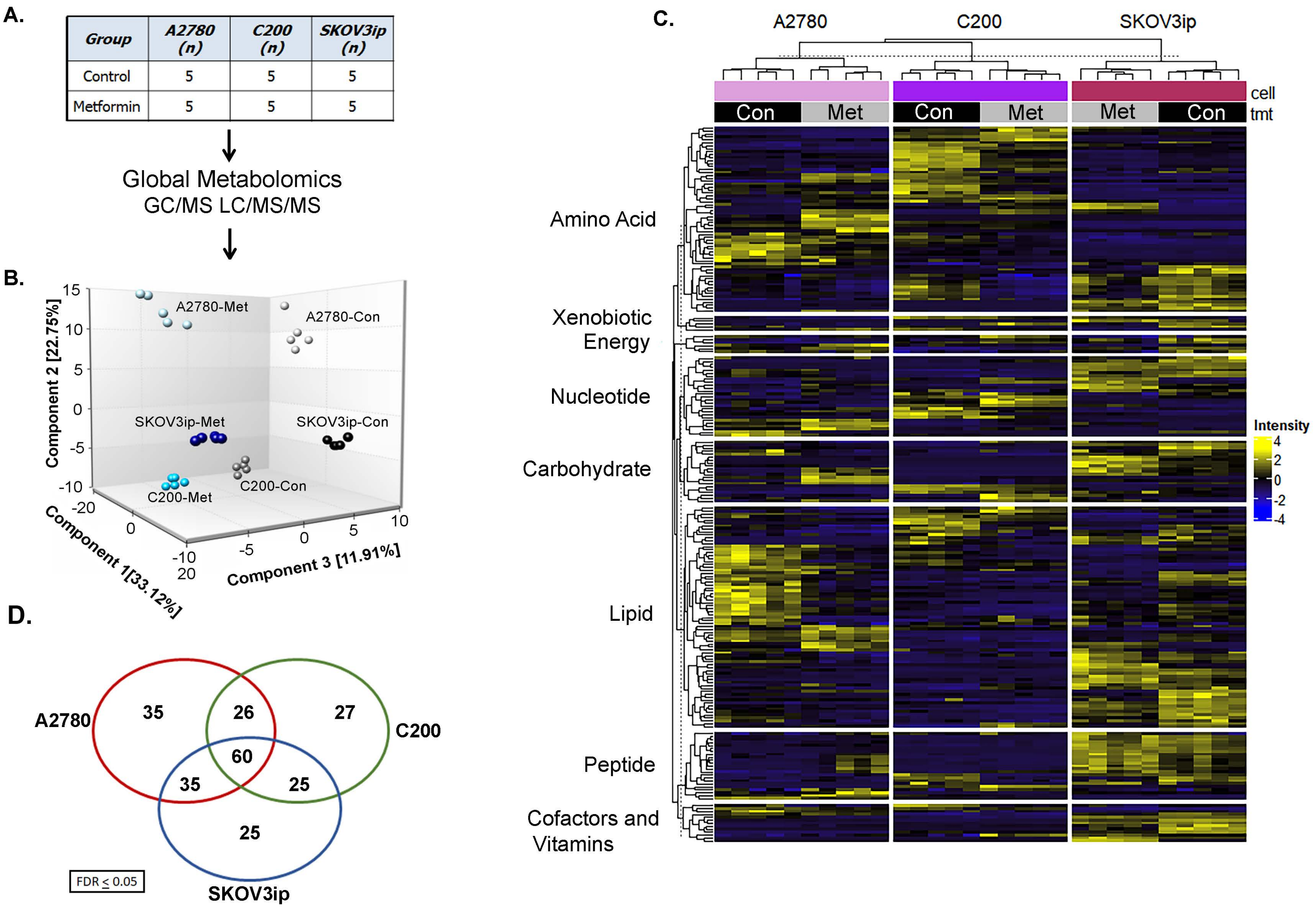

Figure S3: Effect of metformin on glycolysis intermediates is cell line specific.

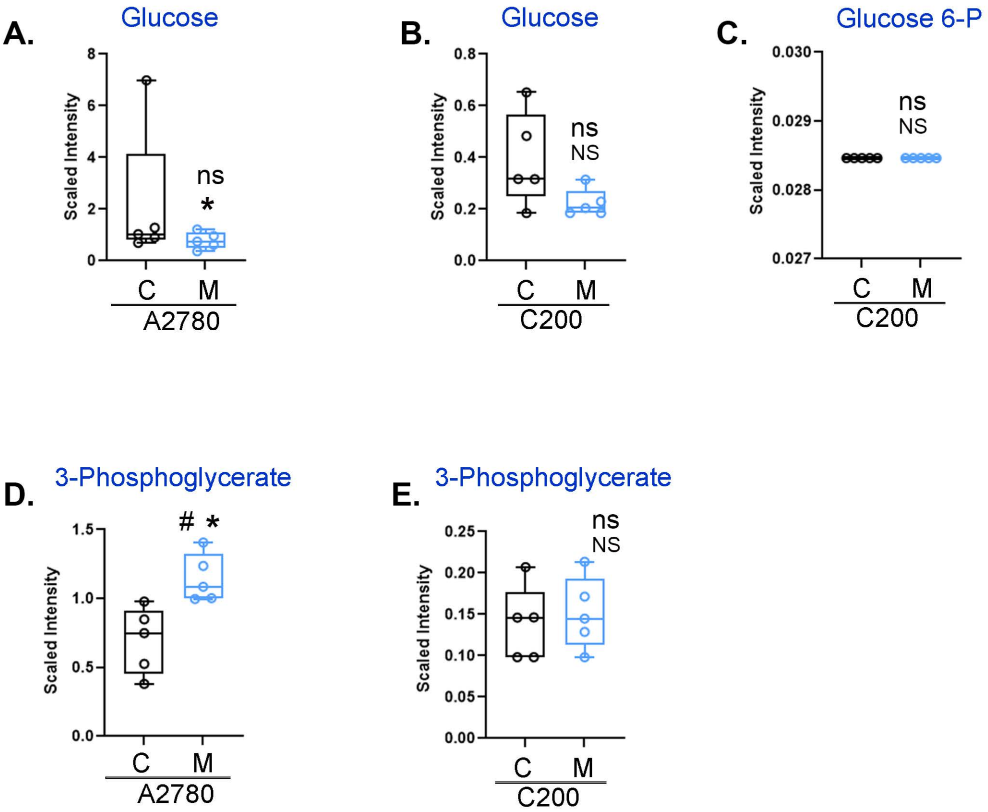

Figure S4 : Effect of metformin on pyrimidine metabolism.

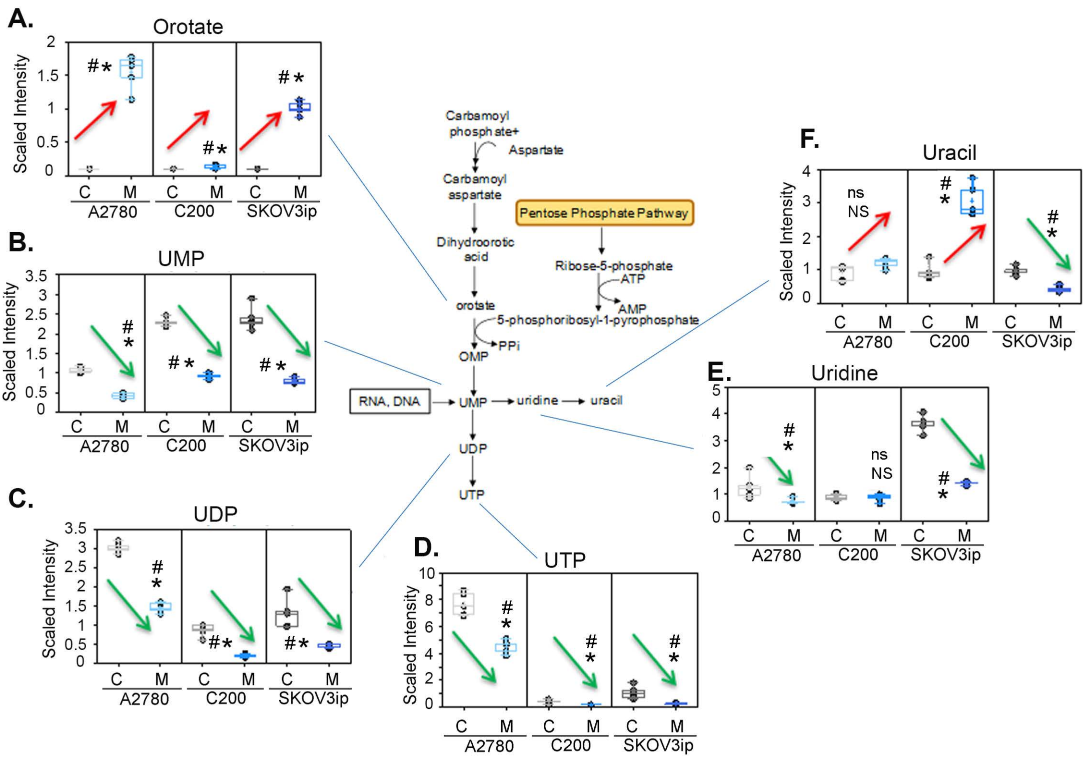

Figure S5: Effect of metformin on polyamine metabolism.

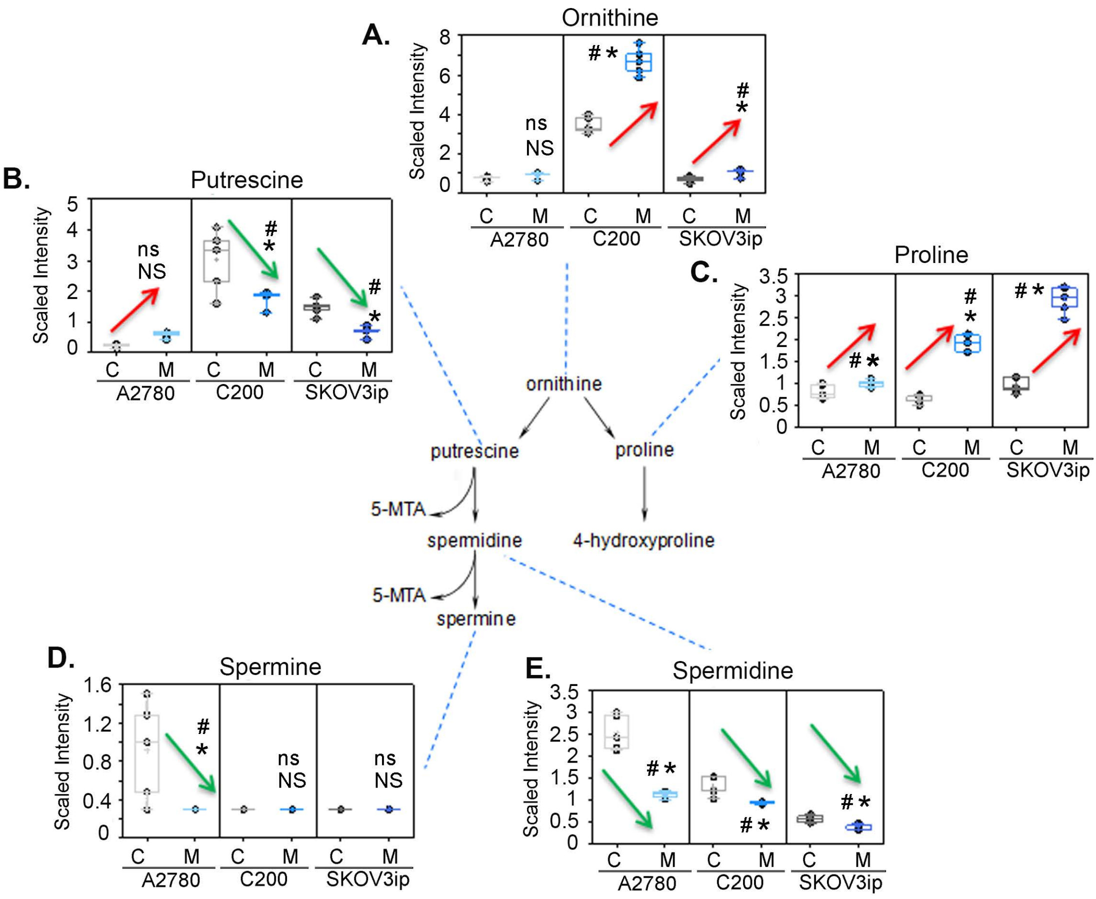

Figure S6: Effect of metformin on essentian FA metabolism.

Biochemical Name	A2780 M/C	C200 M/C	SKOV3ip M/C
linoleate (18:2n6)	2.11	1.17	1.37
linolenate [alpha or gamma; (18:3n3 or 6)]	3.26	1.39	1.21
dihomo-linolenate (20:3n3 or n6)	3.29	3.42	1.49
eicosapentaenoate (EPA; 20:5n3)	2.36	2.35	1.45
docosapentaenoate (n3 DPA; 22:5n3)	2.53	2.25	2.14
docosapentaenoate (n6 DPA; 22:5n6)	2.31	1.23	2.48
docosahexaenoate (DHA; 22:6n3)	1.58	1.28	2.47

Figure S7: EPA and DHA inhibit growth and induce apoptosis in ovarian cancer cells.

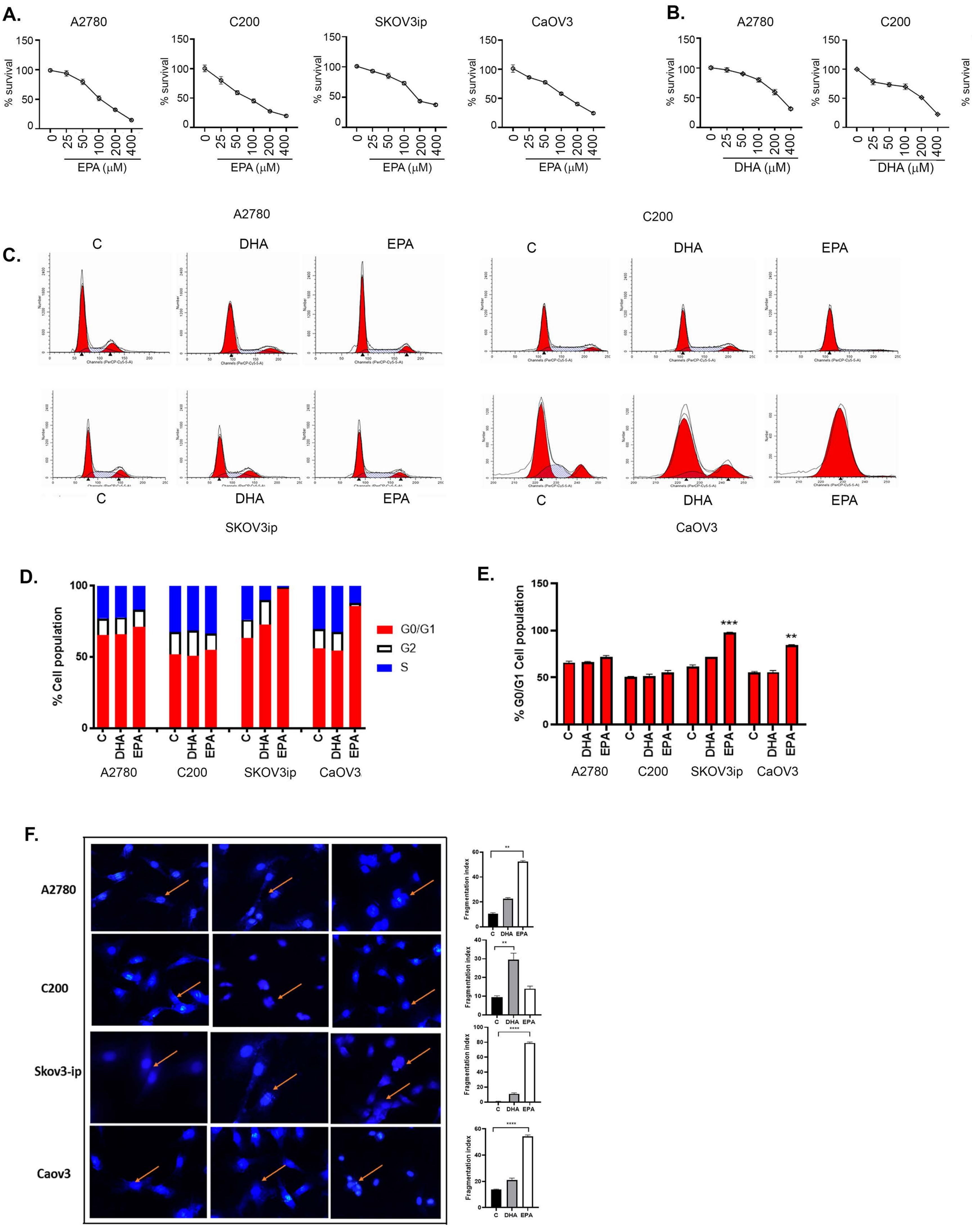

Figure S8: EPA does not induce resolvin E1 in SKOV3ip model.

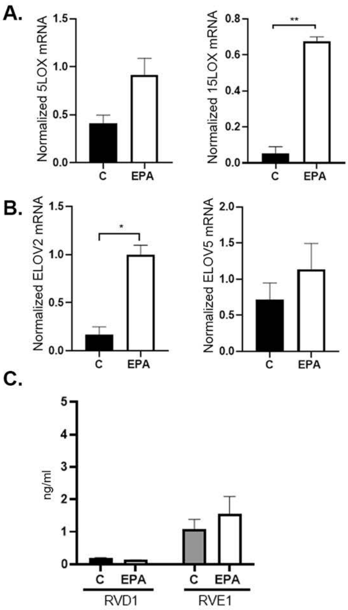

Table S1: List of primers used and sequence

IL-6-F	5'-GAG GAT ACC ACT CCC AAC AGA CC-3'
IL-6-R	5'-AAG TGC ATC ATC GTT GTT CAT ACA-3'
BDH 1 -F	5'-GAA AGT GGT GGA GAT TGT CCG C-3'
BDH1 R	5'-TGT AGG TCT CCA GGC TGG TGA A-3'
Chem 23 F	5’-TTGGCTGAGGACTCATTG-3’
Chem 23 R	5’-CTGATCTTGCACATGGCTGT-3’
Elovl 2 F	5'-CCC TTC GGT TGT CTC ATC TT-3'
Elovl 2 R	5'-CAG GTG GCT CTT GCA TAT CTT-3'
Elovl 5 F	5'-GTG CAC ATT CCC TCT TGG TT-3'
Elovl 5 R	5'-TGG TCC TTC AGG TGG TCT TT-3'
FPR2 F	5'-GCC TTT TGG CTG GTT CCT GTG T-3'
FPR2 R	5'-CAA ATG CAG CGG TCC AAG GCA A-3'
IL 1 B F	5'-TGG AAA AGC GGT TTG TCT TC-3'
IL 1 B R	5'-TAC CAG TTG GGG AAC TCT GC-3'
5 LOX F	5'-GGA GAA CCT GTT CAT CAA CCG C-3'
5 LOX R	5'-CAG GTC TTC CTG CCA GTG ATT C-3'
15 LOX F	5-TACCTGTGGTTGATCGGACA-3
15 LOX R	5’-AGTTCCTCCTCCCTGTGGTT-3
MCP 1 F	5'-GAG AGC TAC AAG AGG ATC ACC A-3'
MCP1 R	5'-GTA TGT CTG GAC CCA TTC CTT C-3'
TNF A F	5'-AAG GAG AAG GCT TGT GAG GTC-3'
TNF A R	5'-TTG TAG AAA GAC CAT GCC TGT G-3'
L27-F	5'-ACA TTG ACG ATG GCA CCT C-3'
L27-F	5'-GCT TGG CGA TCT TCT TCT TG-3'

Table S2: List of Antibodies used for Western Blot.

Antibodies	Source	Identifier	Dilution used
T-AMPK	BD Biosciences	610432	1:1000
P AMPK	Cell signaling	4185S	1:1000
P ACC	Cell signaling	3661L	1:1000
BCL XL	Santacruz	56021	1:1000
Cleaved caspase 3	Cell signaling	9664	1:1000
Cleaved PARP	Cell signaling	9545S	1:1000
Cyclin D1	Santacruz	SC717	1:1000
5 LOX	BD Biosciences	610694	1:2000
15 LOX	Santacruz	133085	1:1000
P 21	Cell Signaling	2946	1:1000
PPAR g	Cell Signaling	27139	1:1000
B actin	Proteintech	Cat# 60008-1-lg	1:3000

Table S3: List of ELISA kits

ELISA	Source	Identifier
IL-6	Biolegend	431301
IL-1 B	Biolegend	432604
MCP-1	Biolegend	432704
TNF- a	Biolegend	430901
Resolvin D1	MyBiosource	MBS058806
Resolvin E1	MyBiosource	MB5755469

Table S4: List of IHC antibodies

Antibody	Source	Identifier
p-AMPK	Cell signaling	2535L
p-ACC	Cell signaling	3661
KI67	Cell signaling	12202S
Cleaved caspase 3	Cell signaling	9579S

Figure S9: Uncropped Western blot images.

Fig 10

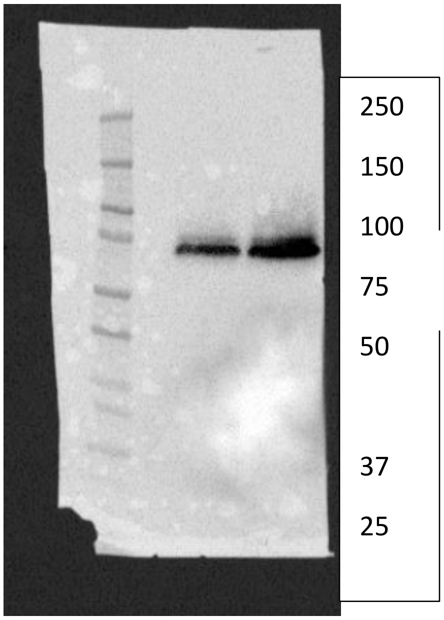

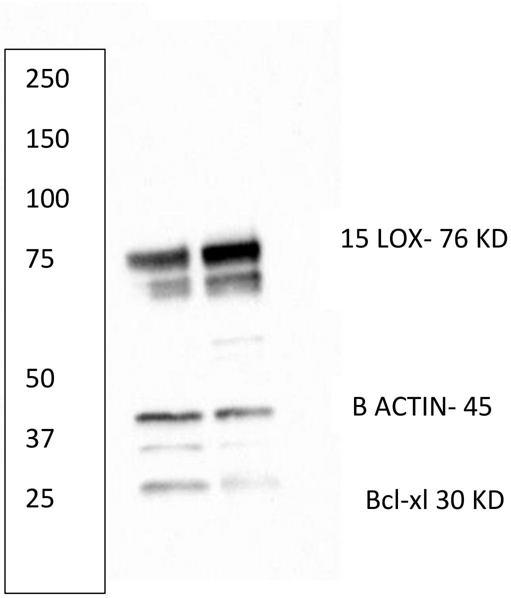

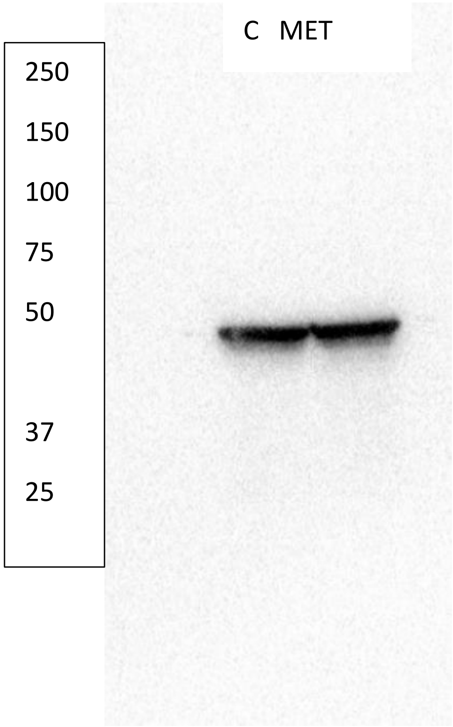

C EPA

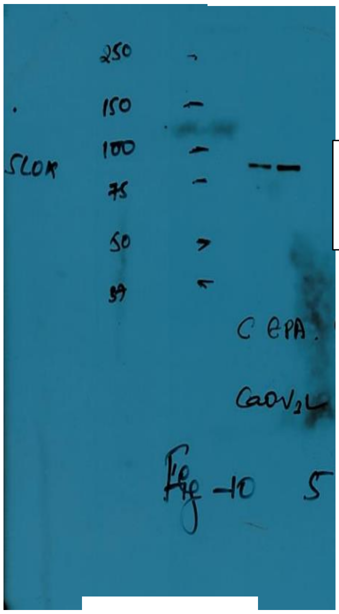

5 LOX (78 KD)

C EPA

C EPA

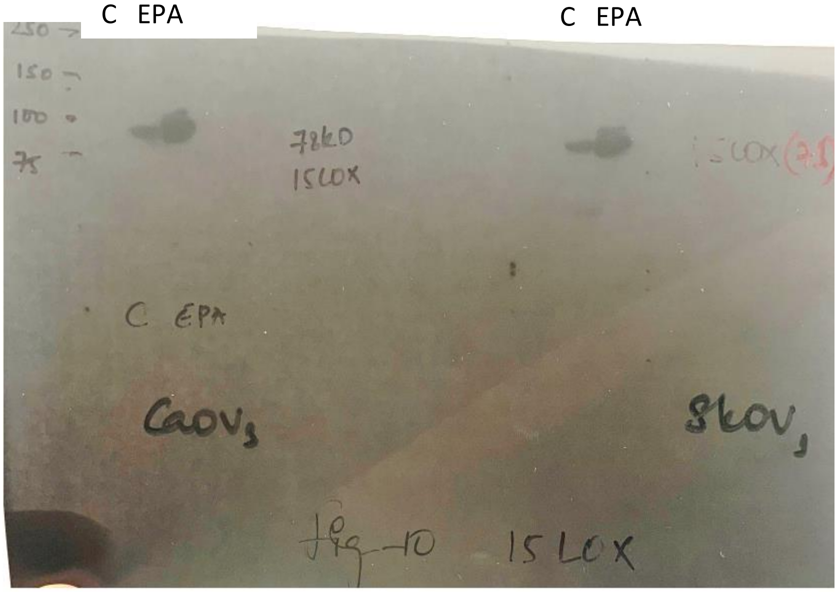

C EPA

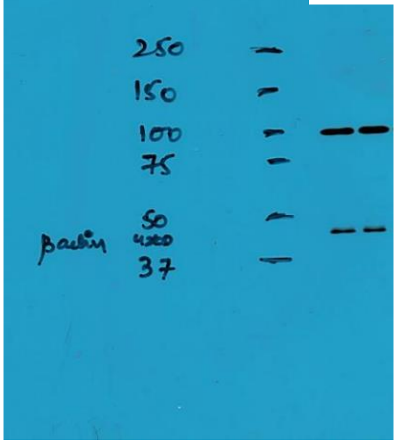

Fig 8

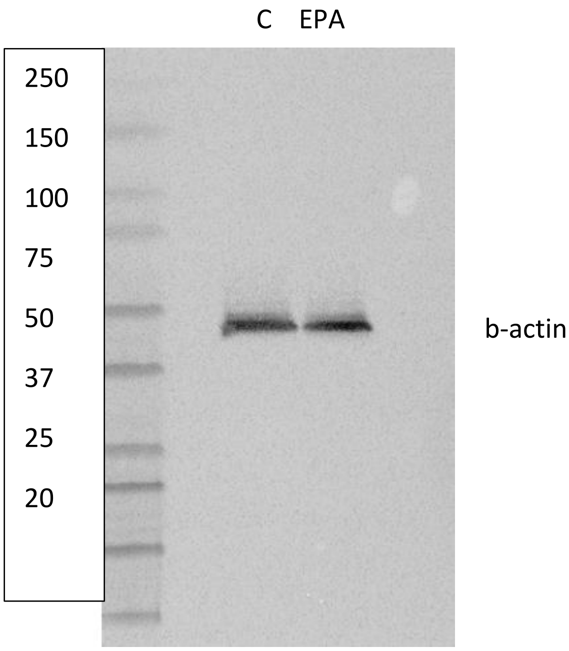
C
EPA
250
150
100
75
50
37
25
20
b-actin

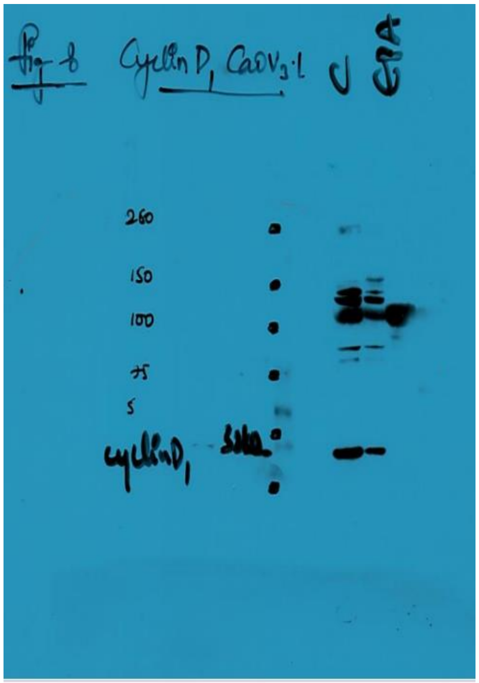
Fig 8
CyclinD1, CaOV3
250
150
100
75

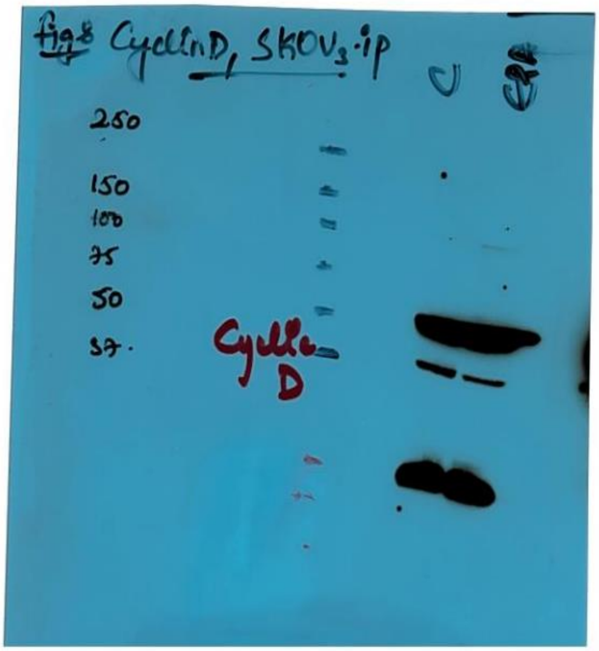
fig 8
CyclinD1, SKOV3·ip
250
150
100
75
50
37
Cyclin D

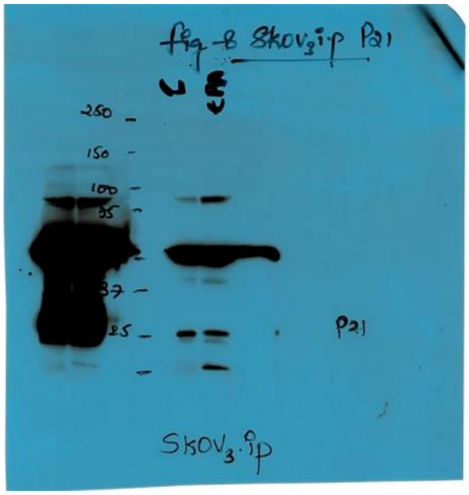
fig-8 Skov3 ip P21
C EPA
250
150
100
75
37
25
P21
SKOV3.ip

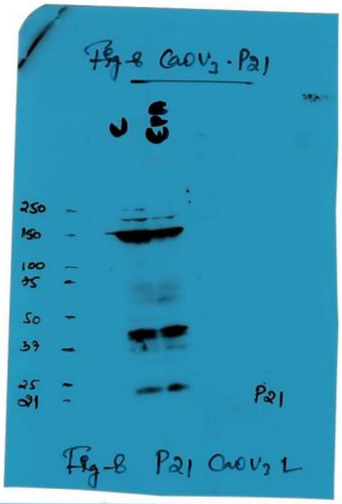
Fig-8 CaOV3 . P21
C EPA
250
150
100
75
50
37
25
21
P21
Fig-8 P21 CaOV3 L

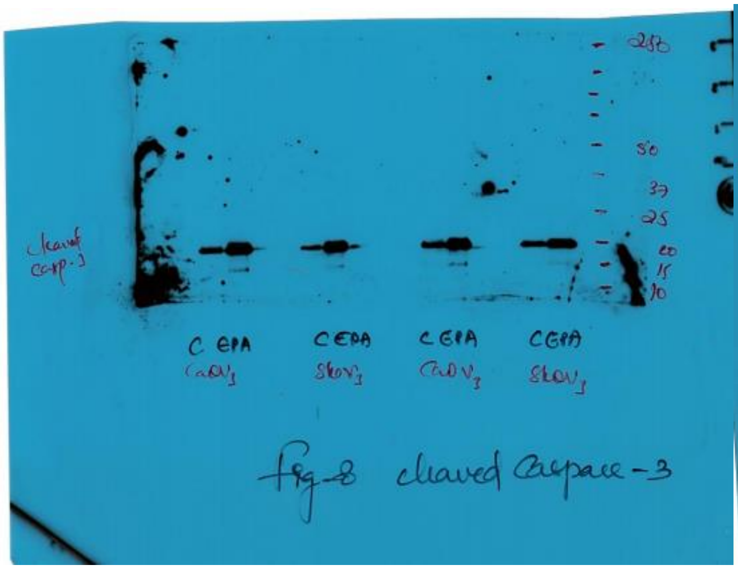
250
50
37
25
20
15
10
cleaved casp-3
C EPA
CaOV3
C EPA
SkOV3
C EPA
CaOV3
C EPA
SkOV3
fig-8 cleaved Caspase-3

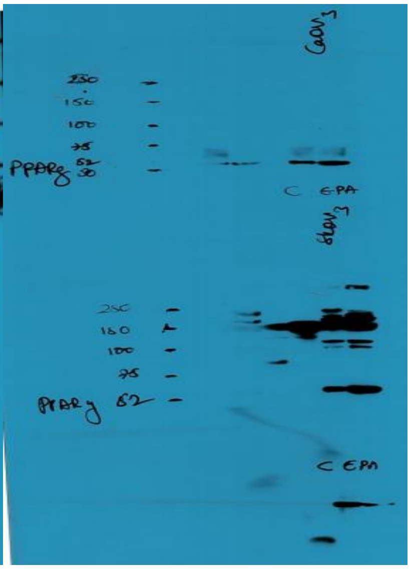
CaOV3
250
150
100
75
PPARg 52
50
C EPA
SkOV3
250
150
100
75
PPARg 52
C EPA

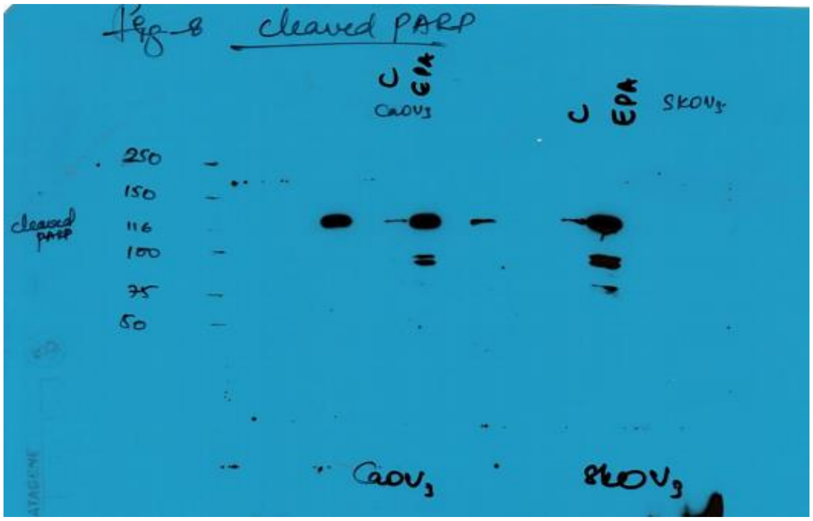
fig-8 cleaved PARP
C EPA
CaOV3
C EPA
SKOV3
250
150
cleaved PARP
116
100
75
50
CaOV3
SkOV3

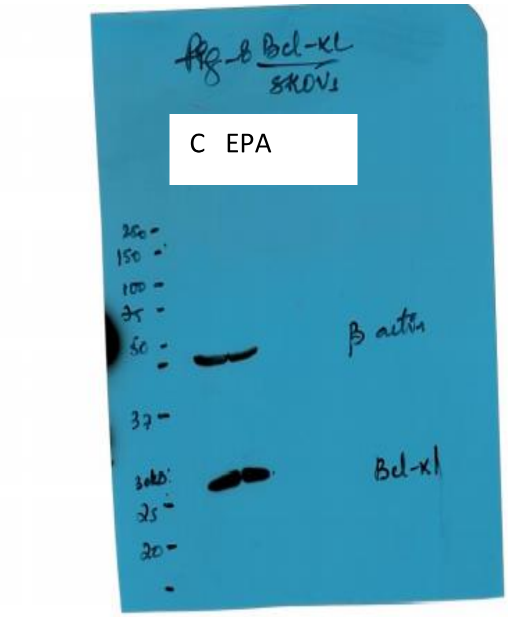
fig-8 Bcl-xL
SKOV3
C EPA
250
150
100
75
50
37
30kD
25
20
β actin
Bcl-xl

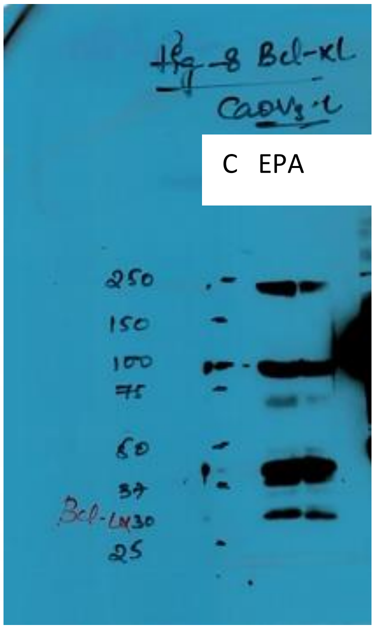
fig-8 Bcl-xL
CaOV3-t
C EPA
250
150
100
75
60
37
Bcl-xL 30
25

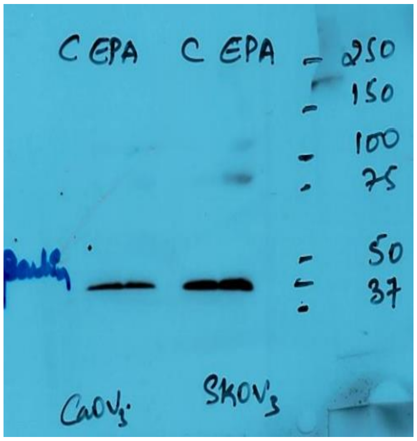
C EPA C EPA
250
150
100
75
50
37
CaOV3
SKOV3

Fig 9

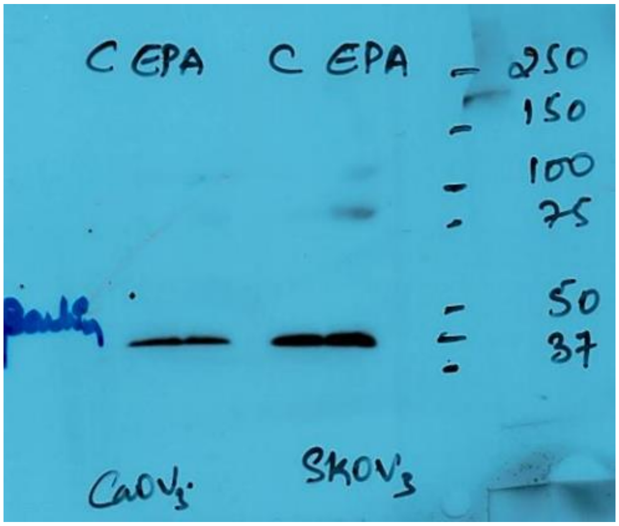
C EPA
C EPA
250
150
100
75
50
37
CaOV3
SKOV3

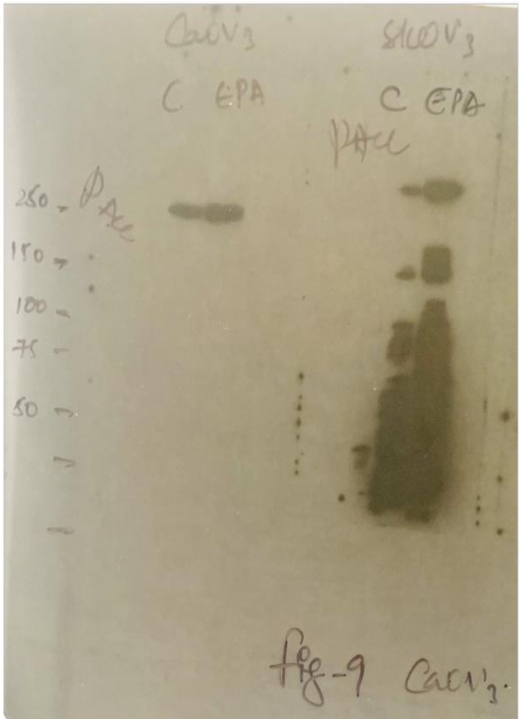
CaOV3
SKOV3
C EPA
C EPA
250
150
100
75
50
fig-9 CaOV3

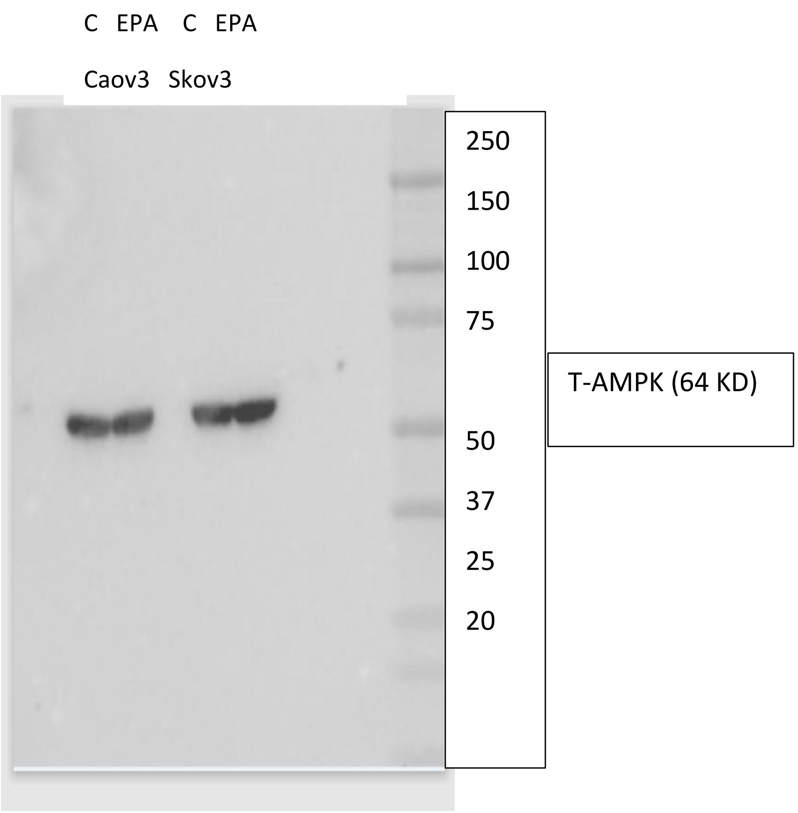
C EPA C EPA
Caov3 Skov3
250
150
100
75
T-AMPK (64 KD)
50
37
25
20

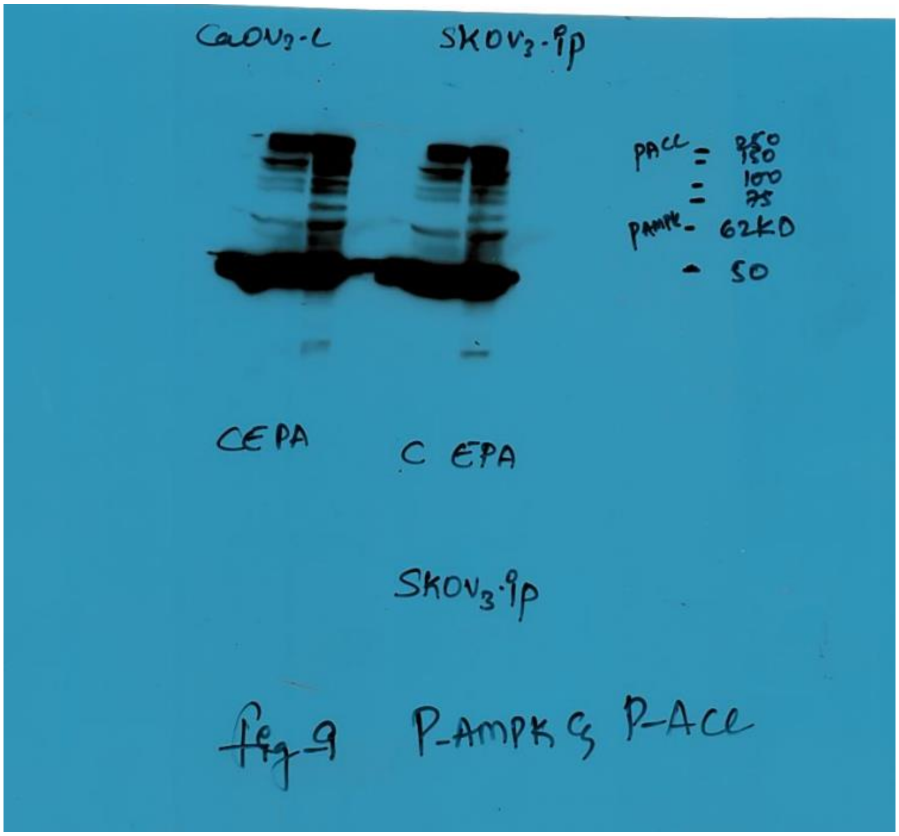
CaOV3-C
SKOV3-ip
PACC
250
150
100
75
pAMPK- 62KD
50
CEPA
C EPA
SKOV3-ip
fig-9 P-AMPK & P-Acc

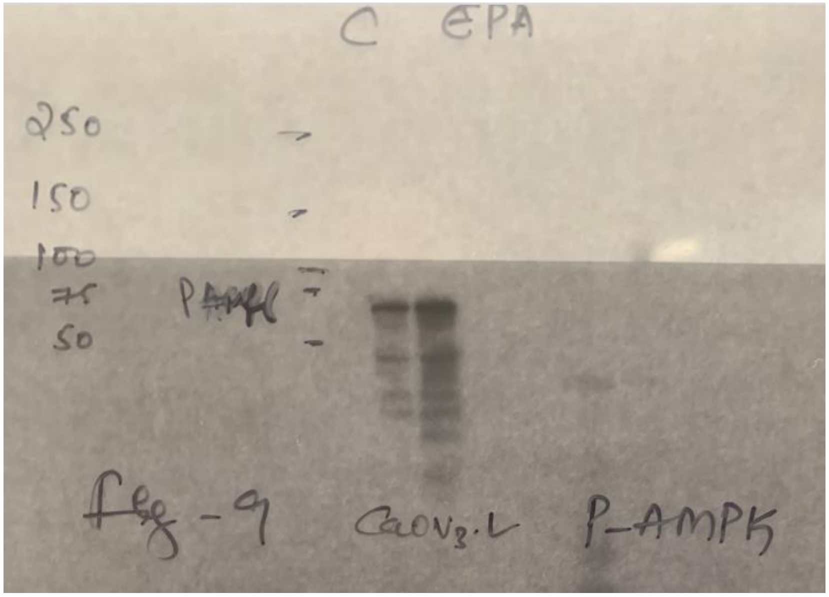
C EPA
250
150
100
75
50
fig-9
P-AMPK

Supplementary Figure Legends

Figure S1: Metformin inhibits ovarian cancer cell proliferation. (A) Cells were counted and plated in 96-well plates (3 x 10^3 cells/well) and treated with indicated concentrations of metformin. Percent cell viability was assessed by MTT performed at 72 hours post-treatment. (B) Cells were counted and plated in 24-well plates (3 x 10^4 cells/well) and treated with indicated concentrations of metformin. Number of alive cells were counted at indicated time point to assess percent increase in cell proliferation. *$p \leq 0.05$, **$p \leq 0.01$, and ***$p \leq 0.001$, respective treated group compared to control.

Figure S2: Global metabolomics analysis of ovarian cancer cells after metformin treatment. (A) A2780, C200 and SKOV3ip cells untreated and treated with metformin 10 Mm (n = 5) for 24 hours were subjected to untargeted global metabolomics. (B) Principal component analysis of A2780, C200 and SKOV3ip cells before and after metformin treatment. (C) Heatmap showing the overall metabolites changes before and after metformin treatment in A2780, C200 and SKOV3ip cells. (D) Statistically significant altered metabolites in both directions A2780, C200 and SKOV3ip cells.

Figure S3: Effect of metformin on pyrimidine metabolic pathway. Scaled intensity of the actual relative levels of various intermediate metabolites of the pyrimidine metabolic pathway in A2780, C200 and SKOV3ip cells are shown before and after metformin treatment: (A) Orotate, (B) UMP; uridine monophosphate, (C) UDP; uridine diphosphate, (D) UTP; uridine triphosphate, (E) uridine and (F) uracil.

Figure S4: Effect of metformin on polyamine metabolic pathway. Scaled intensity of the actual relative levels of various intermediate metabolites of the polyamine metabolic pathway in A2780, C200 and SKOV3ip cells are shown before and after metformin treatment: (A) ornithine, (B) putrescine, (C) proline, (D) spermine, and (E) spermidine.

Figure S5: Effect of metformin on essential FA metabolism. Increased ratio of essential omega-3 fatty acids and their downstream intermediates in A2780, C200 and SKOV3ip cells after metformin treatment.

Figure S6: Enrichment analysis reveal cell specific altered metabolic pathways in response to metformin. Individual enrichment analysis for each cell lines is shown for metabolic pathways that were significantly enriched in the upregulated and downregulated metabolites, individually. The top 6 significantly enriched metabolic pathways that were upregulated and those that were downregulated are shown for A2780 (A and B); C200 (C and D) and SKOV3ip (E and F).

Figure S7: EPA and DHA inhibit growth and induce apoptosis in ovarian cancer cell lines vitro. MTT assay was performed to assess cell viability in A2780, C200, CaOV3 and SKOV3ip cells after treating with various concentrations of (A) EPA or (B) DHA for 48 hours. The untreated control cells were taken as 100% viable to calculate the percent decrease in viability. (C and D) Cell cycle analysis in A2780, C200, CaOV3 and SKOV3ip cells was performed as described in the Materials and Methods after treatment with 50 mM of DHA or EPA for 48 hours. Representative graphs of cell cycle phase distribution obtained from flowcytometry analysis is shown. (D) Bar graph represents relative percent of cells in G0/G1, G2 and S-phase (n = 2) in all the cell lines. (E) Bar graph represents relative percent of cells in phase (n = 2) in all the cell lines in response to EPA or DHA. (F) Hoechst staining was performed to assess nuclear fragmentation index in A2780, C200, CaOV3 and SKOV3ip cells after treatment with 50 mM of DHA or EPA for 72 hours. Bar graph represents percent of nuclear fragments compared to total number of cells and presented as percent fragmented fragments (n = 3).

Figure S8: EPA has no effect on formation of resolving E1 in SKOV3ip model. (A) mRNA expression of 5-LOX and 15-LOX in pooled SKOV3ip tumors (n = 3) treated with EPA or vehicle control. (B) mRNA expression of ELOVL2 and ELOVL 5 in pooled SKOV3ip tumors (n = 3) treated with EPA or vehicle control. (C) RvE1 protein levels measured in plasma isolated from SKOV3ip tumors treated with EPA or vehicle control (n = 4).

Figure S9: Uncropped Western blot images.

Table S1: List and sequence of the primers used in the study.

Table S2: List of the antibodies with catalogue number and dilution used.

Table S3: List of the ELISA kits with catalogue number used in the study.

Table S4: List of the antibodies used in immunohistochemistry with catalogue number.
